# Synergistic roles of DYRK1A and GATA1 in trisomy 21 megakaryopoiesis

**DOI:** 10.1172/jci.insight.172851

**Published:** 2023-10-31

**Authors:** Ying Ting Sit, Kaoru Takasaki, Hyun Hyung An, Yan Xiao, Christian Hurtz, Peter A. Gearhart, Zhe Zhang, Paul Gadue, Deborah L. French, Stella T. Chou

**Affiliations:** 1 Division of Hematology, Department of Pediatrics, Children’s Hospital of Philadelphia, Philadelphia, Pennsylvania, USA.; 2 Fels Cancer Institute for Personalized Medicine, Lewis Katz School of Medicine, Temple University, Philadelphia, Pennsylvania, USA.; 3 Deparment of Obstetrics and Gynecology, Pennsylvania Hospital, University of Pennsylvania Health System, Philadelphia, Pennsylvania, USA.; 4 Department of Biomedical Informatics and; 5 Center for Cellular and Molecular Therapeutics, Children’s Hospital of Philadelphia, Philadelphia, Pennsylvania, USA.; 6 Department of Pathology and Laboratory Medicine, Perelman School of Medicine at the University of Pennsylvania, Philadelphia, Pennsylvania, USA.

**Keywords:** Hematology, Leukemias, iPS cells

## Abstract

Patients with Down syndrome (DS), or trisomy 21 (T21), are at increased risk of transient abnormal myelopoiesis (TAM) and acute megakaryoblastic leukemia (ML-DS). Both TAM and ML-DS require prenatal somatic mutations in *GATA1*, resulting in the truncated isoform GATA1s. The mechanism by which individual chromosome 21 (HSA21) genes synergize with GATA1s for leukemic transformation is challenging to study, in part due to limited human cell models with wild-type GATA1 (wtGATA1) or GATA1s. HSA21-encoded *DYRK1A* is overexpressed in ML-DS and may be a therapeutic target. To determine how DYRK1A influences hematopoiesis in concert with GATA1s, we used gene editing to disrupt all 3 alleles of *DYRK1A* in isogenic T21 induced pluripotent stem cells (iPSCs) with and without the *GATA1s* mutation. Unexpectedly, hematopoietic differentiation revealed that DYRK1A loss combined with GATA1s leads to increased megakaryocyte proliferation and decreased maturation. This proliferative phenotype was associated with upregulation of D-type cyclins and hyperphosphorylation of Rb to allow E2F release and derepression of its downstream targets. Notably, DYRK1A loss had no effect in T21 iPSCs or megakaryocytes with wtGATA1. These surprising results suggest that DYRK1A and GATA1 may synergistically restrain megakaryocyte proliferation in T21 and that DYRK1A inhibition may not be a therapeutic option for GATA1s-associated leukemias.

## Introduction

Children with Down syndrome (DS), or trisomy 21 (T21), are at increased risk for transient abnormal myelopoiesis (TAM) and acute megakaryoblastic leukemia of DS (ML-DS) (1, 2). TAM is a neonatal preleukemic condition that arises in the fetus from a unique genetic interaction between T21 and the exclusive expression of GATA1s, the N-terminus–truncated isoform of the key hematopoietic transcription factor GATA-binding protein 1 (GATA1). The blasts of TAM and ML-DS are both characterized by somatic mutations in GATA1 that result in GATA1s (3, 4), but ML-DS blasts additionally acquire “third-hit” mutations, typically in an epigenetic regulator or cohesin complex member (5, 6). Notably, germline GATA1s mutations in individuals without T21 result in congenital anemia, thrombocytopenia, and/or neutropenia but are not associated with leukemia (7, 8), confirming the requirement of both GATA1s and T21 to promote leukemia.

The cell cycle is precisely controlled in hematopoietic development. GATA1 has been shown to repress cell cycle progression and proliferation, and to promote terminal differentiation in hematopoietic cells by preventing the binding of the transcriptional activator E2Fs to their downstream targets (9–11). The Rb/E2F pathway is critical for cell cycle regulation and is typically repressed by GATA1; however, as the GATA1 N-terminus is critical for this interaction, GATA1s is unable to repress activator E2Fs (9–11). GATA1 also inhibits GATA2 (GATA-binding protein 2), a hematopoietic transcription factor that is crucial for hematopoietic stem cell (HSC) and megakaryocyte expansion, and frequently overexpressed in ML-DS (12). Without the N-terminal domain, GATA1s is unable to properly downregulate GATA2, leading to hyperproliferation of HSCs and megakaryocytes (13, 14).

How genes on chromosome 21 (HSA21) alter hematopoiesis remains incompletely understood, but a candidate region of 8.35 Mb has been implicated in increased risk for TAM and ML-DS by phenotype correlation of individuals with rare segmental T21 (15). The gene *DYRK1A* (dual-specificity tyrosine-phosphorylation-regulated kinase 1A) is located in that region and is overexpressed in ML-DS and TAM samples by 1.5- to 3-fold compared with non-DS ML (12, 16, 17). While a number of studies have indicated that DYRK1A is involved in neurologic, cardiac, and immunological disorders associated with DS (18–20), only a few studies have characterized its role in hematopoiesis (16, 21–23).

In nonhematopoietic cells, DYRK1A has been shown to regulate the cell cycle through its kinase activity via several mechanisms (24–26). DYRK1A phosphorylates and subsequently destabilizes D-type cyclins to induce cell cycle arrest in neurons and cardiomyocytes (19, 20). In human T21 skin fibroblasts, increased dosage of DYRK1A phosphorylates and destabilizes cyclin D1 to prolong the G_1_ phase and induce cell cycle arrest (27). DYRK1A also phosphorylates Lin52, a component of the dimerization partner, RB-like, E2F, and multi-vulval class B (DREAM) complex that regulates cell cycle–dependent gene expression and inhibits cell cycle entry (28).

In contrast, in ex vivo assays using euploid murine bone marrow cells, *Dyrk1a* overexpression induced expansion of CD41^+^ megakaryocytes through dysregulation of nuclear factor of activated T cells (NFAT) transcription factors, which was further enhanced in *Gata1s*-mutant progenitors (16). In murine lymphoid cells, loss of DYRK1A kinase activity resulted in cyclin D3 stabilization, thereby promoting G_0_ to G_1_/S phase progression; however, this also led to late cell cycle (G_2_-M) blockade, ultimately resulting in decreased cell division (22, 23). Taken together, these studies suggest a cell context–dependent role for DYRK1A in hematopoiesis and a potential role in T21 leukemic transformation.

Murine DS models have provided important insights into the pathophysiology of DS phenotypes, but only partially recapitulate the hematopoietic abnormalities associated with T21 (29–31). Notably, DS mice do not develop leukemia when crossed with GATA1s mice, underscoring the importance of studying the function of DYRK1A in human cell models (13, 16, 29–31). The role of DYRK1A in cell proliferation is dosage and cellular context dependent (25, 27), and may also be species specific. Here, we studied the role of DYRK1A in a human T21 induced pluripotent stem cell (iPSC) model and found distinct roles of DYRK1A in isogenic DS hematopoietic progenitors and committed megakaryocytes in the context of either wild-type GATA1 (wtGATA1) or GATA1s. Our studies reveal that DYRK1A and wtGATA1 synergistically restrain megakaryocyte proliferation, and in the presence of *GATA1s* mutations, DYRK1A loss unexpectedly exacerbates the expansion of immature megakaryocytes. These findings were surprising since DYRK1A overexpression has been shown to promote murine megakaryocyte expansion, while pharmacologic inhibition enhances maturation of human neonatal megakaryocytes (16, 21), underscoring the cell context– and species-specific effects of this HSA21-encoded gene.

## Results

### DYRK1A is overexpressed in human T21 cells.

**Figure 1:**
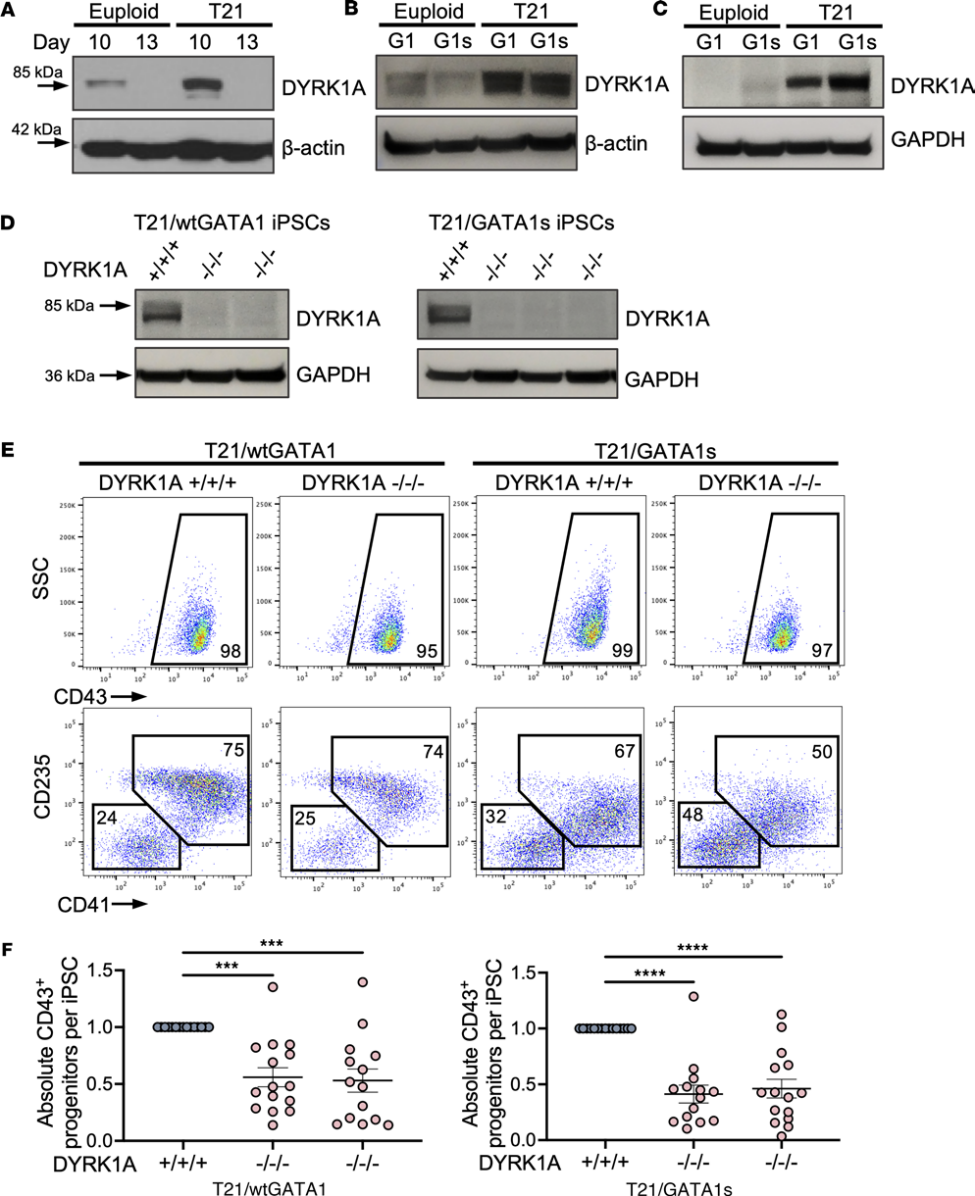
DYRK1A loss results in aberrant hematopoietic progenitor potential. (**A**) Western blot for DYRK1A expression in CD61-selected ex vivo fetal liver–derived megakaryocytes, (**B**) undifferentiated iPSCs, and (**C**) iPSC-derived megakaryocytes on day 6 of liquid culture from euploid or T21 iPSCs, with wild-type GATA1 (G1) or GATA1 short (G1s). Percentage CD41^+^CD42^+^ cells from lanes 1 to 4: 70%, 68%, 76%, and 78%, respectively. (**D**) Western blot for DYRK1A expression in DYRK1A-untargeted and -targeted T21/wtGATA1 and T21/GATA1s iPSCs. Each lane represents an individual iPSC clone. (**E**) Flow cytometric analysis of hematopoietic progenitors on day 7 from EB differentiation of T21/wtGATA1 or T21/GATA1s iPSCs, with DYRK1A WT (+/+/+) or knockout (–/–/–). (**F**) Absolute CD43^+^ progenitor yield on day 7 of EB differentiation, normalized to starting DYRK1A^+/+/+^ iPSC number. Each column represents an individual iPSC clone. *n* = 14–15 independent experiments per clone. Data represent the mean ± SEM. Statistical significance was determined by ordinary 1-way ANOVA. ***P ≤ 0.001, ****P ≤ 0.0001.

To assess the role of DYRK1A in the pathogenesis of ML-DS arising from fetal progenitors, we measured its expression in euploid and T21 fetal liver–derived megakaryocytes, as well as in undifferentiated euploid and T21 iPSCs and iPSC-derived megakaryocytes with and without the GATA1s mutation. Compared with euploid cells, DYRK1A was overexpressed in T21 cells in all 3 tissue types (Figure 1, A–C, and Supplemental Figure 1; supplemental material available online with this article; https://doi.org/10.1172/jci.insight.172851DS1). Notably, in iPSC-derived megakaryocytes, DYRK1A expression was further increased in cells with GATA1s (T21/GATA1s) compared with wtGATA1 (Figure 1C and Supplemental Figure 1C). While the levels of DYRK1A expression do vary over the course of differentiation at all time points, T21/GATA1s megakaryocytes expressed increased DYRK1A compared with T21/wtGATA1 (Supplemental Figure 10B). Although T21 typically results in small increases in HSA21 gene expression, this marked increase in DYRK1A in T21 tissues, and specifically in megakaryocytes expressing GATA1s, prompted our studies to focus on DYRK1A. CRISPR/Cas9 gene editing was used to knock out all isoforms of human DYRK1A in isogenic T21 iPSC lines with wtGATA1 or GATA1s that previously had been generated by our laboratory (32, 33) (Supplemental Figure 2 and Supplemental Table 1). DYRK1A loss (DYRK1A^–/–/–^) was confirmed by Western blotting (Figure 1D) and clones analyzed are listed in Supplemental Table 2.

### DYRK1A loss alters hematopoietic progenitor cells.

To evaluate the effects of DYRK1A deficiency on the blood-forming capacities of T21/wtGATA1 and T21/GATA1s iPSCs, we performed hematopoietic differentiation by embryoid body (EB) formation (34, 35). On day 7 of EB differentiation, both CD43^+^CD41^+^CD235^+^ and CD43^+^CD41^–^CD235^–^ hematopoietic progenitor populations were produced from DYRK1A^+/+/+^ and DYRK1A^–/–/–^ lines (Figure 1E). Consistent with prior studies, both CD43^+^CD41^+^CD235^+^ and CD43^+^CD41^–^CD235^–^ populations were multipotent (36). Of note, T21/GATA1s progenitors did not generate erythroid cells (32), and consistently showed lower CD235 mean fluorescence intensity (MFI) compared with T21/wtGATA1 progenitors (Supplemental Figure 3). DYRK1A^–/–/–^ resulted in lower CD41 and CD235 MFI regardless of GATA1 status (Supplemental Figure 3), and in the T21/GATA1s/DYRK1A^–/–/–^ clones, the CD43^+^ day 7 progenitor population was skewed toward CD41^–^CD235^–^ (Figure 1E). The absolute number of day 7 CD43^+^ progenitors generated per iPSC undergoing differentiation was decreased in both T21/wtGATA1 and T21/GATA1s lines when DYRK1A was disrupted (Figure 1F). Together, these data suggest that loss of DYRK1A alters hematopoietic progenitor cell production in the setting of T21.

### DYRK1A loss enhances megakaryocyte and myeloid expansion in T21 progenitors with GATA1s.

**Figure 2:**
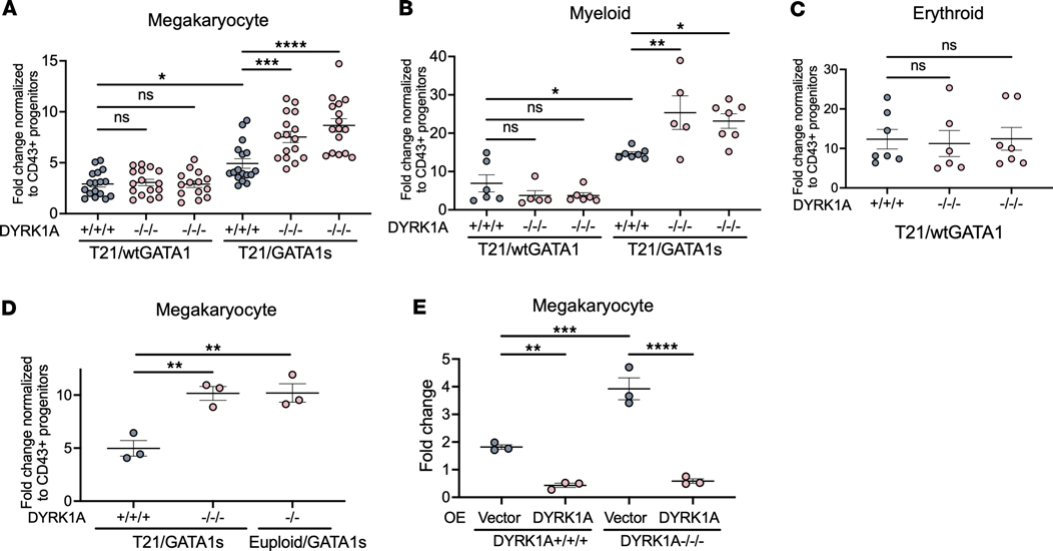
DYRK1A loss selectively enhances megakaryocyte and myeloid potential in the context of T21/GATA1s. Fold change of indicated lineages from T21/wtGATA1 or T21/GATA1s iPSC–derived CD43^+^ hematopoietic progenitors with DYRK1A WT (+/+/+) or knockout (–/–/–), differentiated in liquid culture for 6 days to support (**A**) megakaryocyte (CD41^+^CD42^+^), (**B**) myeloid (CD45^+^CD18^+^), or (**C**) erythroid (CD235^+^CD71^+^) cell growth. Each column represents an individual iPSC clone. *n* = 14–17, 5–7, and 6–7 independent experiments per clone for panels **A**, **B**, and **C**, respectively. (**D**) Megakaryocyte (CD41^+^CD42^+^) fold-change growth from euploid/GATA1s/DYRK1A^–/–^ CD43^+^ progenitors on day 6 of megakaryocyte liquid culture, compared with T21/GATA1s with DYRK1A WT (+/+/+) or knockout (–/–/–). *n* = 3 independent experiments per clone. (**E**) Megakaryocyte fold-change growth from T21/GATA1s CD43^+^ progenitors with DYRK1A WT (+/+/+) or knockout (–/–/–) transduced with lentivirus containing the HMD vector to express enhanced green fluorescence protein (GFP) or HMD-DYRK1A to express GFP and DYRK1A, and subsequently cultured for 6 days. Fold change normalized to day 2 GFP^+^. *n* = 3 independent experiments. Data represent the mean ± SEM. Statistical significance was determined by ordinary 1-way ANOVA. NS, not significant. *P ≤ 0.05; **P ≤ 0.01; ***P ≤ 0.001; ****P ≤ 0.0001.

To determine the effects of DYRK1A loss on erythroid, megakaryocyte, and myeloid lineages, CD43^+^ progenitors from day 7 of hematopoietic differentiation of T21/wtGATA1 and T21/GATA1s iPSCs with DYRK1A^+/+/+^ or DYRK1A^–/–/–^ were assayed by lineage-specific liquid cultures. Consistent with our previous findings (32), T21/GATA1s/DYRK1A^+/+/+^ progenitors showed enhanced megakaryocytic and myeloid growth capacity compared with T21/wtGATA1/DYRK1A^+/+/+^ progenitors, as measured by fold-change growth (gray dots; Figure 2, A and B). In the context of T21/wtGATA1, DYRK1A^+/+/+^ and DYRK1A^–/–/–^ progenitors differentiated into megakaryocytic (CD41^+^CD42^+^), myeloid (CD45^+^CD18^+^), or erythroid (CD71^+^CD235^+^) lineages with similar frequency and proliferative capacity (Figure 2, A–C). In contrast, loss of DYRK1A in T21/GATA1s enhanced the fold-change growth of megakaryocyte and myeloid cells from total day 7 CD43^+^ progenitors (Figure 2, A and B), as well as flow cytometry–purified CD43^+^CD41^+^CD235^+^ and CD43^+^CD41^–^CD235^–^ cells (data not shown). Independently generated iPSC clones from another T21 individual with TAM confirmed the decrease in absolute number of CD43^+^ progenitors and increased megakaryocyte fold-change growth with DYRK1A loss (Supplemental Figure 4, A and B). While generating clones with 1- or 2-allele *DYRK1A* disruption was challenging given the high efficiency of the CRISPR/Cas9 guide RNAs (gRNAs), analysis of a clone with 2 disrupted alleles demonstrated an intermediate phenotype with decreased progenitor production and increased megakaryocyte expansion, but to a lesser extent compared with 3-allele *DYRK1A*–knockout clones (Supplemental Figure 5).

We next used colony-forming assays to determine lineage bias of CD43^+^ progenitors from T21/wtGATA1 and T21/GATA1s lines with DYRK1A^+/+/+^ or DYRK1A^–/–/–^ to produce megakaryocyte, myeloid, and erythroid colonies. Day 7 CD43^+^ (CD43^+^CD41^+^CD235^+^ and CD43^+^CD41^–^CD235^–^ that are CD45^–^CD18^–^) progenitor cells were seeded into colony-forming megakaryocyte (CFU-Mk) assays containing thrombopoietin (TPO), interleukin-3 (IL-3), and IL-6, or methylcellulose assays with stem cell factor (SCF), IL-3, erythropoietin (EPO), and granulocyte-macrophage colony–stimulating factor (GM-CSF) to support myeloid and erythroid colony formation. We observed no difference in megakaryocyte, myeloid, or erythroid colony formation among day 7 CD43^+^ progenitors from T21/wtGATA1 or T21/GATA1s with DYRK1A^+/+/+^ or DYRK1A^–/–/–^ (Supplemental Figure 6, A and B). Taken together with the fold-change growth of T21/GATA1s/DYRK1A^–/–/–^ CD43^+^ progenitors in megakaryocyte and myeloid liquid cultures, DYRK1A loss combined with T21/GATA1s may enhance megakaryocyte and myeloid cell proliferation but does not alter their cell fate or lineage potential.

While gene targeting the T21/GATA1s iPSCs, we identified a clone that lost the redundant copy of HSA21. Interestingly, DYRK1A knockout in euploid/GATA1s iPSC–derived progenitors demonstrated enhanced megakaryocyte proliferation similar to the DYRK1A loss in the context of T21, suggesting the interplay between DYRK1A and GATA1s may not require the extra copy of HSA21 (Figure 2D). Conversely, DYRK1A overexpression by lentiviral transduction decreased megakaryocyte growth from both T21/GATA1s/DYRK1A^+/+/+^ and T21/GATA1s/DYRK1A^–/–/–^ CD43^+^ hematopoietic progenitors (Figure 2E). The restricted growth with *DYRK1A* overexpression contrasts with murine studies where *Dyrk1a* overexpression in euploid wtGATA1 or GATA1s bone marrow cells induced expansion of megakaryocytes (16). Our results suggest a species-specific role of DYRK1A in human progenitor cells, where DYRK1A restrains megakaryocyte expansion and its loss combined with GATA1s further exacerbates the enhanced megakaryocyte growth observed with *GATA1s* mutations.

### DYRK1A loss enhances megakaryocyte proliferation in the context of T21/GATA1s.

**Figure 3:**
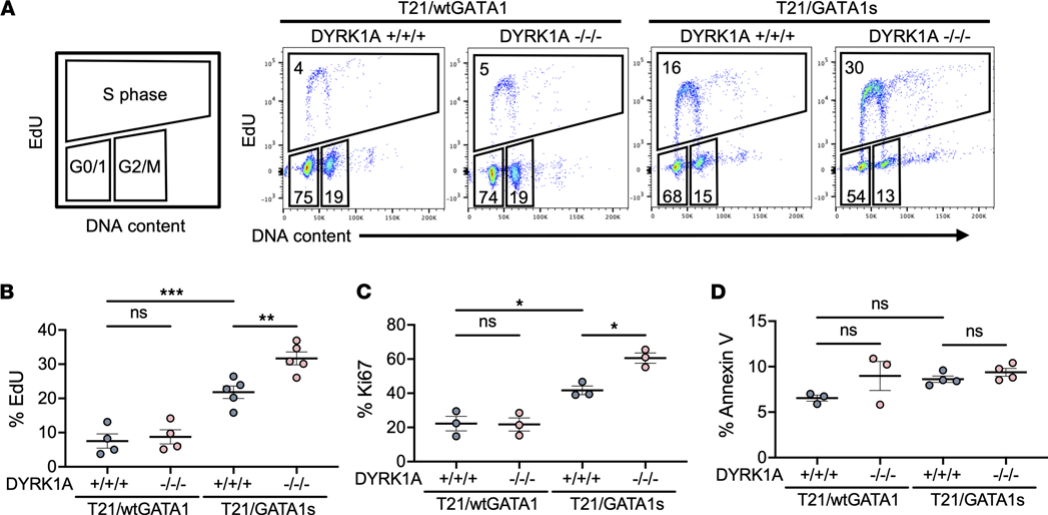
DYRK1A loss selectively enhances megakaryocyte proliferation in the context of T21/GATA1s. (**A**) Representative EdU flow cytometric analysis of CD41^+^CD42b^+^ megakaryocytes differentiated from T21/wtGATA1 and T21/GATA1s iPSCs with DYRK1A WT (+/+/+) or knockout (–/–/–). (**B**) Percentage EdU-, (**C**) percentage Ki67-, and (**D**) percentage Annexin V–positive CD41^+^CD42b^+^ megakaryocytes on day 5 to 6 of megakaryocyte culture of T21/wtGATA1 and T21/GATA1s CD43^+^ progenitors with DYRK1A WT (+/+/+) or knockout (–/–/–). *n* = 3–5 independent experiments per genotype. Data represent the mean ± SEM. Statistical significance was determined by ordinary 1-way ANOVA. NS, not significant. *P ≤ 0.05; **P ≤ 0.01; ***P ≤ 0.001.

DYRK1A has diverse roles in the cell cycle by regulating the balance between cell cycle entry and quiescence, which is dependent on cellular context (37). We assayed cell cycle stages in T21/wtGATA1 and T21/GATA1s CD41^+^CD42b^+^ megakaryocytes with DYRK1A^+/+/+^ or DYRK1A^–/–/–^ by measuring EdU DNA incorporation on days 5 to 6 of culture (Figure 3A). We previously demonstrated that GATA1s enhances megakaryocyte expansion compared with wtGATA1 irrespective of HSA21 status (32). On average, T21/GATA1s/DYRK1A^+/+/+^ megakaryocytes incorporated EdU in 22% of cells, representing those in S phase, compared with 7.5% in T21/wtGATA1/DYRK1A^+/+/+^ megakaryocytes (Figure 3B). The number of megakaryocytes in S phase increased further to an average of 32% with T21/GATA1s/DYRK1A^–/–/–^, while the percentage of T21/wtGATA1 megakaryocytes in S phase was not significantly changed with DYRK1A loss (Figure 3, A and B). Consistent with EdU assays, T21/GATA1s/DYRK1A^+/+/+^ megakaryocytes showed an increased proportion of Ki67^+^ mitotically active cells compared with T21/wtGATA1/DYRK1A^+/+/+^, which was further increased with DYRK1A loss in T21/GATA1s but not T21/wtGATA1 cells (Figure 3C). DYRK1A loss had no effect on apoptosis in either T21/wtGATA1 or T21/GATA1s megakaryocytes, as measured by flow cytometry for Annexin V (Figure 3D). These findings support a model where DYRK1A loss specifically increases megakaryocyte proliferation in the context of T21/GATA1s.

### DYRK1A loss impairs maturation of T21/GATA1s megakaryocytes.

**Figure 4:**
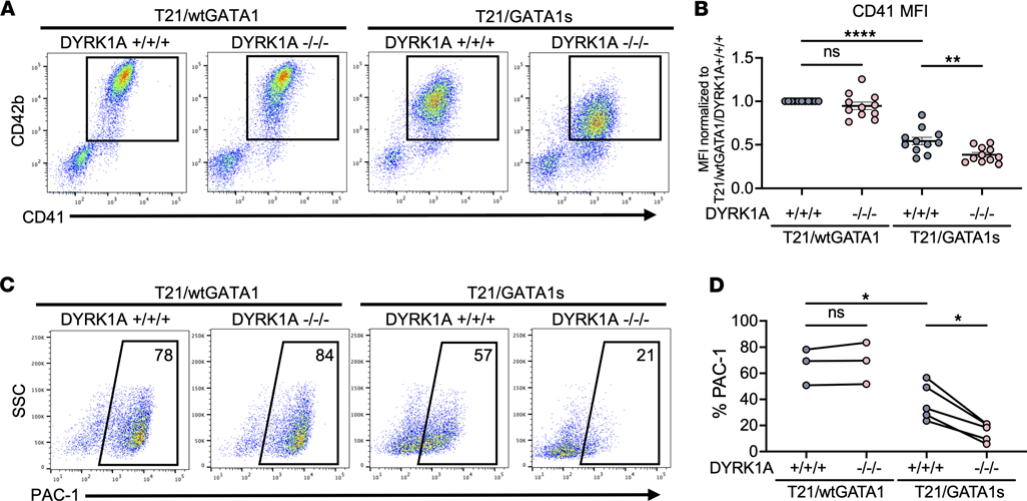
DYRK1A loss selectively impairs megakaryocyte activation and maturation in the context of T21/GATA1s. (**A**) Representative and (**B**) composite CD41 mean fluorescence intensity (MFI) of megakaryocytes from T21/wtGATA1 and T21/GATA1s iPSCs with DYRK1A WT (+/+/+) or knockout (–/–/–), assayed on day 5 or 6 of megakaryocyte liquid culture. *n* = 11 independent experiments per genotype. (**C**) Representative and (**D**) composite flow cytometric analyses for PAC-1 after thrombin stimulation of megakaryocytes. *n* = 3–5 independent experiments per genotype. Data represent the mean ± SEM. Statistical significance was determined by ordinary 1-way ANOVA. NS, not significant. *P ≤ 0.05, **P ≤ 0.01, ****P ≤ 0.0001.

Since proliferation and maturation are often uncoupled, we performed characterization of T21/wtGATA1 and T21/GATA1s megakaryocytes with DYRK1A^+/+/+^ or DYRK1A^–/–/–^ to determine whether DYRK1A loss impairs maturation. We observed several features in T21/GATA1s/DYRK1A^–/–/–^ megakaryocytes consistent with an impaired capacity to mature. First, we found a significant decrease in CD41 and CD42b MFI in T21/GATA1s/DYRK1A^+/+/+^ megakaryocytes that was further reduced by DYRK1A loss, consistent with an immature phenotype (Figure 4, A and B). DYRK1A loss in T21/GATA1s megakaryocytes was associated with decreased forward scatter (FSC) and side scatter (SSC), as measured by flow cytometry (Supplemental Figure 7A); smaller size and less granularity are consistent with less mature megakaryocytes. Lentiviral overexpression of *DYRK1A* rescued this phenotype, with increased CD41 MFI in the megakaryocytes differentiated from transduced T21/GATA1s/DYRK1A^+/+/+^ and T21/GATA1s/DYRK1A^–/–/–^ CD43^+^ progenitors (Supplemental Figure 7B).

To test the effect of DYRK1A loss on megakaryocyte function, we analyzed their responsiveness to stimulation with thrombin. Activation with thrombin results in a conformational change in the surface integrin αIIbβ3 receptors that enables the binding of the conformation-specific monoclonal antibody, PAC-1 (38). All megakaryocytes showed minimal basal PAC-1 binding, demonstrating no spontaneous preactivation of these cells in culture. Upon thrombin stimulation, T21/GATA1s/DYRK1A^+/+/+^ megakaryocytes demonstrated an approximately 30% lower level of PAC-1 binding following thrombin activation compared with T21/wtGATA1/DYRK1A^+/+/+^ (average 38% vs. 66%, *P* ≤ 0.05). DYRK1A loss resulted in a significant further decrease in PAC-1 binding in T21/GATA1s megakaryocytes (average 15%, *P* ≤ 0.05), providing functional evidence of impaired megakaryocyte maturation, while it had no effect on T21/wtGATA1 megakaryocytes (Figure 4, C and D).

### T21/GATA1s/DYRK1A^–/–/–^ megakaryocytes have a proliferative gene signature.

**Figure 5:**
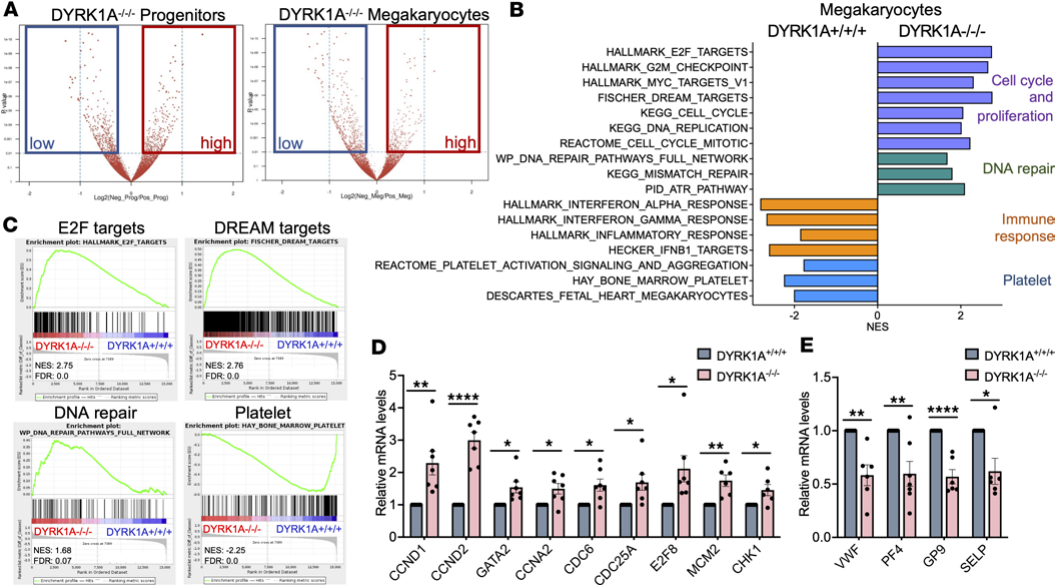
T21/GATA1s/DYRK1A^–/–/–^ megakaryocytes demonstrate enhanced cell proliferation and decreased platelet signaling. Flow cytometry–sorted T21/GATA1s/DYRK1A^+/+/+^ and DYRK1A^–/–/–^ CD41^+^CD43^+^CD235^+^ progenitors or CD41^+^CD42b^+^ megakaryocytes on day 4 of megakaryocyte liquid culture for RNA analyses. (**A**) Volcano plots showing differential gene expression for T21/GATA1s/DYRK1A^–/–/–^ compared with DYRK1A^+/+/+^. Each dot represents 1 gene, with gating of the dot reflecting the clustering information for each gene; dots gated in red squares are genes that are upregulated compared with DYRK1A^+/+/+^; dots gated in blue square are genes that are downregulated compared with DYRK1A^+/+/+^. (**B**) Up- and downregulated pathways from gene set enrichment analysis (GSEA) of RNA-seq data. (**C**) GSEA for indicated pathways comparing DYRK1A^–/–/–^ to DYRK1A^+/+/+^. NES, normalized enrichment score; FDR, false discovery rate. (**D**) Relative mRNA expression levels for *CCND1*, *CCND2*, *GATA2*, and E2F target genes. (**E**) Relative mRNA expression levels for megakaryocyte-related genes. *n* = 5–7 independent experiments per genotype. Data represent the mean ± SEM. Statistical significance was determined by 2-tailed, unpaired *t* test. *P ≤ 0.05, **P ≤ 0.01, ****P ≤ 0.0001.

To investigate the mechanism underlying DYRK1A’s effect on T21/GATA1s hematopoietic cells, we performed RNA sequencing (RNA-seq) of flow cytometry–purified CD43^+^CD41^+^CD235^+^ progenitors and CD41^+^CD42b^+^ megakaryocytes from T21/GATA1s iPSCs with DYRK1A^+/+/+^ or DYRK1A^–/–/–^. Among the 21,451 genes examined, 15,159 were expressed in at least 1 cell type. Overall, 1,380 genes were differentially expressed in hematopoietic progenitors, with 654 genes upregulated and 726 downregulated with DYRK1A^–/–/–^. In megakaryocytes, 1,187 genes were differentially expressed, with 444 genes upregulated and 743 downregulated with DYRK1A^–/–/–^ (Benjamini-Hochberg FDR < 0.25) (Figure 5A and Supplemental Figure 8).

Gene set enrichment analysis (GSEA) of T21/GATA1s progenitors with DYRK1A^–/–/–^ compared with DYRK1A^+/+/+^ showed increased enrichment of pathways related to endothelial and CD34^+^ cells consistent with the abnormal progenitor production we observed, and decreased enrichment of interferon signaling genes, which have an important role in the self-renewal, quiescence, and differentiation of HSCs (Supplemental Figure 9) (39). Consistent with our observed phenotype of enhanced proliferation in T21/GATA1s/DYRK1A^–/–/–^ megakaryocytes, GSEA in megakaryocytes but not progenitors identified enrichment of genes related to cell cycle, cell proliferation, and cell division (Figure 5B). E2F targets were enriched in T21/GATA1s/DYRK1A^–/–/–^ megakaryocytes (Figure 5C), suggesting DYRK1A loss in addition to GATA1s derepresses E2F (9–11). DYRK1A is also important for the formation of the DREAM complex, for repression of DREAM target genes, and for induction of quiescence (28, 40). Genes typically repressed by the DREAM complex were enriched in T21/GATA1s/DYRK1A^–/–/–^ megakaryocytes (Figure 5C), suggesting DYRK1A loss results in DREAM complex dissociation. The DREAM complex typically represses activator E2Fs (E2F1, -2, and -3), but with its dissociation these bind to downstream targets to promote cell cycle entry. DNA damage repair responses that are required for highly proliferating cancer cells to maintain chromosome stability were also enriched (Figure 5, B and C). Consistent with impaired maturation, T21/GATA1s/DYRK1A^–/–/–^ megakaryocytes showed decreased enrichment for genes involved in platelet activation and aggregation (Figure 5, B and C).

Among all genes, *CCND2* (cyclin D2) and the hematopoietic transcription factor *GATA2* were among the top 5 most upregulated in T21/GATA1s/DYRK1A^–/–/–^ megakaryocytes besides replication-dependent histones. Quantitative reverse transcriptase PCR (qRT-PCR) confirmed upregulation of these genes (Figure 5D). Other E2F-related or target genes, such as *CCND1* (cyclin D1), *CCNA2* (cyclin A2), *CDC6*, *CDC25A*, *E2F8*, *MCM2*, and *CHK1* were upregulated in T21/GATA1s/DYRK1A^–/–/–^ megakaryocytes, while platelet and megakaryocyte pathway–related genes, including *VWF*, *PF4*, *GP9*, and *SELP* were downregulated (Figure 5E). Overall, these data are consistent with our findings that T21/GATA1s/DYRK1A^–/–/–^ megakaryocytes show enhanced proliferation but reduced maturation and activation potential.

### DYRK1A loss increases expression of cell cycle and proliferative proteins in T21/GATA1s megakaryocytes.

**Figure 6:**
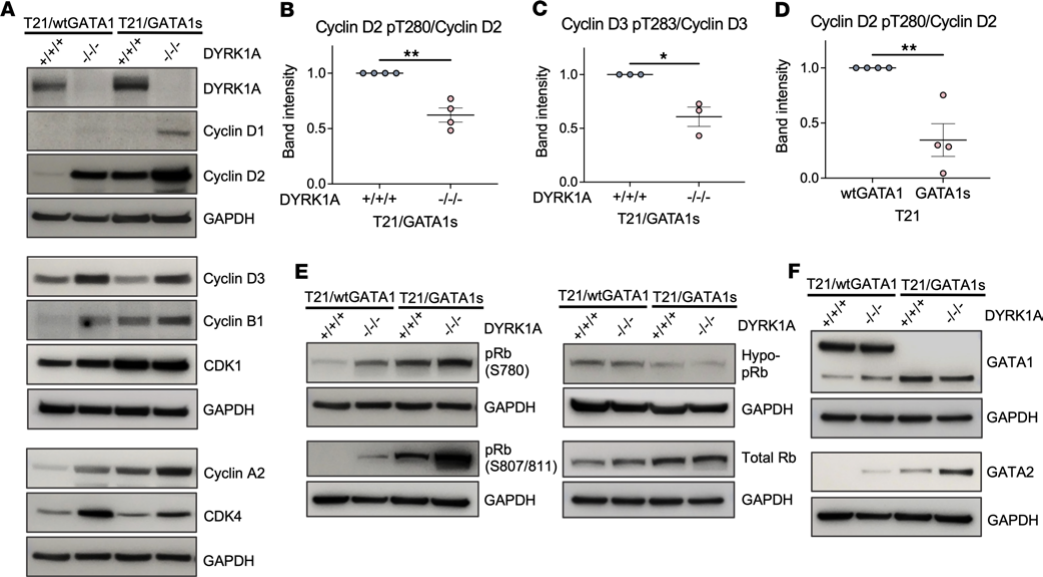
Increased expression of proliferative genes in T21/GATA1s/DYRK1A^–/–/–^ megakaryocytes. (**A**) Representative Western blot analysis of day 4 T21/wtGATA1 or T21/GATA1s megakaryocytes with DYRK1A WT (+/+/+) or knockout (–/–/–) for DYRK1A and cell cycle–related proteins. (**B** and **D**) Phosphorylation of cyclin D2 (pT280) and (**C**) cyclin D3 (pT283) quantified by Western blot band intensity and normalized to total cyclin D2 or D3 expression. *n* = 3–4 independent experiments per genotype. Data represent the mean ± SEM. Statistical significance was determined by 2-tailed, unpaired *t* test. *P ≤ 0.05, **P ≤ 0.01. (**E**) Representative Western blot analysis of day 4 T21/wtGATA1 or T21/GATA1s megakaryocytes with DYRK1A WT (+/+/+) or knockout (–/–/–) for phosphorylated (pRb), hypophosphorylated (hypo-pRb), and total Rb, and (**F**) GATA1 and GATA2.

DYRK1A has been shown to regulate the cell cycle and inhibit proliferation through the cyclin D/CDK-Rb/E2F pathway (19, 37). Since E2F targets, D-type cyclins, and *GATA2* mRNA levels were upregulated in T21/GATA1s/DYRK1A^–/–/–^ megakaryocytes, we compared protein expression of cyclin, Rb, and GATA in T21/wtGATA1 and T21/GATA1s megakaryocytes with DYRK1A^+/+/+^ or DYRK1A^–/–/–^. Without DYRK1A manipulation, many cell cycle–related genes were already expressed at a higher level in T21/GATA1s megakaryocytes compared with T21/wtGATA1, including cyclin D2, cyclin B1, CDK1, and cyclin A2 (Figure 6A), consistent with a proliferative state. Comparable levels of cyclin D1, cyclin D3, and CDK4 were observed. In both T21/wtGATA1 and T21/GATA1s megakaryocytes, DYRK1A loss resulted in upregulation of cell cycle–related genes, including all 3 D-type cyclins (D1, D2, and D3), cyclin B1, cyclin A2, and CDK4 (Figure 6A). Western blot analysis of megakaryocytes from an independent patient-derived iPSC line confirmed the upregulation of cell cycle–related genes in association with T21/GATA1s and DYRK1A^–/–/–^ (Supplemental Figure 10, A and B).

In cardiomyocytes and lymphocytes, DYRK1A promotes quiescence through phosphorylation of cyclin D2 at Thr280 and cyclin D3 at Thr283, respectively, leading to their ubiquitination and degradation (19, 22). While total cyclin D2 and D3 expression was increased in T21/wtGATA1 and T21/GATA1s megakaryocytes with DYRK1A^–/–/–^, the proportion that was phosphorylated, i.e., cyclin D2 pT280 and cyclin D3 pT283, was decreased, which would facilitate cell cycle entry (Figure 6, B and C, and Supplemental Figure 11). Of note, the proportion of phosphorylated cyclin D2 (pT280) to total cyclin D2 was lower in T21/GATA1s/DYRK1A^+/+/+^ compared with T21/wtGATA1/DYRK1A^+/+/+^ megakaryocytes (Figure 6D and Supplemental Figure 11), consistent with cyclin D2 stabilization and a proliferative state.

When cells are stimulated to enter the cell cycle, cyclin D–CDK4/6 complexes phosphorylate Rb (41). In T21/GATA1s megakaryocytes, higher levels of phosphorylated Rb were observed compared with T21/wtGATA1 (Figure 6E). DYRK1A loss resulted in a further increase in Rb phosphorylation and a decrease in hypophosphorylated Rb (Figure 6E), which lead to release of activator E2Fs and increased cell cycle entry.

While GATA1 and GATA2 expression overlap in megakaryocytes, these well-described GATA switches can alter transcription and cell fate (42, 43). DYRK1A knockout did not alter full-length GATA1 or GATA1s protein levels in iPSC-derived megakaryocytes (Figure 6F). However, *GATA2*, which is associated with megakaryocyte expansion and is overexpressed in ML-DS samples (12), was among the top upregulated genes in the RNA-seq analysis in T21/GATA1s/DYRK1A^–/–/–^ megakaryocytes. GATA2 expression was increased in T21/GATA1s/DYRK1A^+/+/+^ megakaryocytes compared with T21/wtGATA1/DYRK1A^+/+/+^ (Figure 6F and Supplemental Figure 10B). We observed a striking increase in GATA2 expression with DYRK1A loss in T21/GATA1s megakaryocytes (Figure 6F), consistent with enhanced proliferation of immature megakaryocytes and dysregulated GATA2 expression that has been linked to myeloid leukemogenesis (43) and ML-DS (12).

## Discussion

Children with DS have an approximately 150-fold increased risk of developing acute myeloid leukemia (44), with the majority having a *GATA1s* mutation along with additional mutations in genes encoding cohesin complex members or epigenetic regulators (5, 6). Despite significant work, it remains unclear how genes on chromosome 21 cooperate with these additional mutations to cause disease. The HSA21-encoded gene *DYRK1A* is associated with several malignancies, both tumor promoting and tumor suppressing (37), and has been specifically implicated in ML-DS pathogenesis in a murine model (16). Here, we elucidate the role of DYRK1A in human DS hematopoietic progenitors and committed megakaryocytes in the context of T21 and GATA1s.

**Figure 7:**
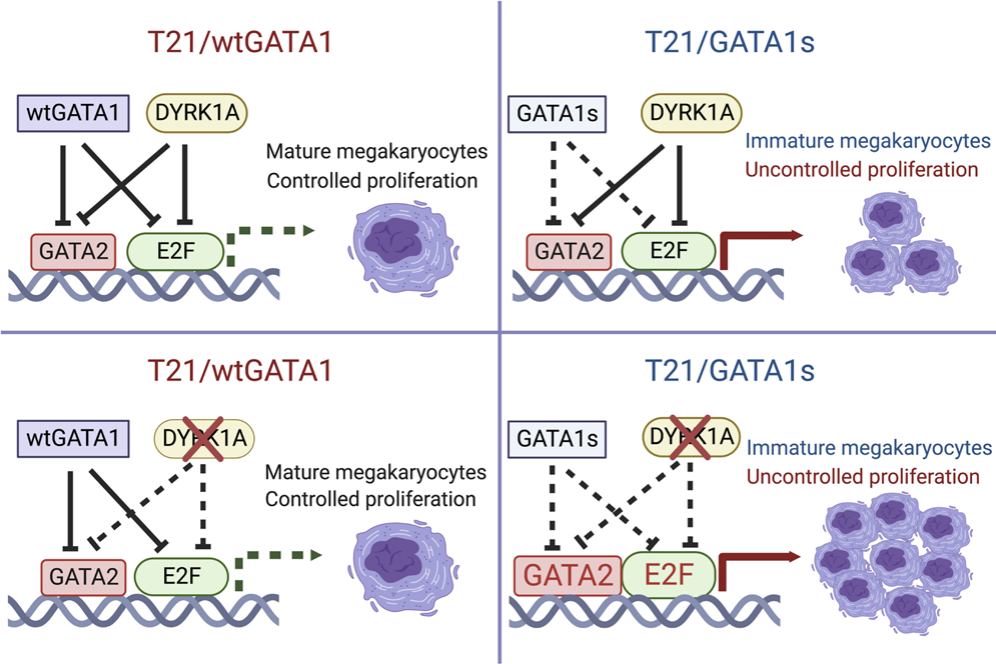
Model of GATA1 and DYRK1A synergistically controlling megakaryocyte growth. DYRK1A and wtGATA1 both repress the E2F and GATA2 pathways to control T21 megakaryocyte growth and maturation. DYRK1A loss in T21/wtGATA1 cells does not affect megakaryocyte proliferation or maturation. In the presence of *GATA1* mutations leading to the N-terminus–truncated GATA1s, excess growth of immature megakaryocytes results from derepression of GATA2 and E2F. This is further exacerbated by DYRK1A loss that leads to aberrant megakaryopoiesis, resulting in proliferative, immature megakaryocytes.

We used patient-derived T21 iPSCs to demonstrate that cell proliferation is precisely controlled by both wtGATA1 and DYRK1A to orchestrate megakaryocyte proliferation and maturation (Figure 7). Prior work has demonstrated that wtGATA1 restricts IGF-mediated activation of the E2F transcriptional network that coordinates cell proliferation and differentiation (10). In nonproliferating cells, GATA1, E2F, and hypophosphorlyated Rb form a tricomplex to inhibit the E2F pathway and subsequent cell cycle entry (9). DYRK1A loss in T21/wtGATA1 megakaryocytes leads to enhanced cell cycle–related gene expression but does not result in increased proliferation since activator E2Fs and GATA2 are sufficiently inhibited by wtGATA1.

The highly conserved wtGATA1 motif LXCXE that spans amino acid positions 81–83 is required for formation of the GATA1-Rb-E2F complex; GATA1s is missing this motif and without formation of this complex, E2F is derepressed and drives unchecked cell proliferation (9, 45). In T21/GATA1s cells, GATA1s fails to inhibit E2F and GATA2, leading to increased megakaryocyte proliferation and impaired maturation. Consistent with this, E2F target genes are significantly upregulated in human TAM and ML-DS samples (10). In our iPSC model, DYRK1A knockout in T21/GATA1s megakaryocytes is associated with a further increase in GATA2 expression, upregulation of all 3 D-type cyclins (D1, D2, and D3), and phosphorylation of Rb to allow E2F release and derepression of its downstream targets. Taken together, these results show that DYRK1A loss combined with GATA1s leads to activated cell cycle entry, uncontrolled proliferation, and impaired maturation (Figure 7). Of note, T21/GATA1s megakaryocytes with 2 of 3 *DYRK1A* alleles disrupted showed an intermediate cell expansion phenotype compared with T21/GATA1s/DYRK1A^–/–/–^, but did not show significantly impaired megakaryocyte maturation by PAC-1 binding (Supplemental Figure 5 and data not shown).

While significant research has characterized the antiproliferative role of DYRK1A in nonhematopoietic cells (19, 20, 27, 46), a smaller body of work suggests DYRK1A promotes megakaryopoiesis and cell proliferation (16, 21). Retroviral overexpression of *Dyrk1a* in ex vivo assays using euploid murine bone marrow cells induced an expansion of CD41^+^ megakaryocytes that was modestly enhanced with *Gata1s* (16). Moreover, *Dyrk1a* knockdown in trisomic *Ts1Rhr* and *Gata1s/Ts1Rhr* bone marrow cells decreased the megakaryocytic expansion observed in these trisomic mice. Notably, the same studies performed in murine fetal liver cells led to only slight differences, supporting developmentally specific effects of *Dyrk1a* dosage. The species-specific divergence of our findings that DYRK1A restrains megakaryocyte proliferation in human hematopoietic cells versus a proliferative role in murine megakaryopoiesis is reminiscent of contrasting observations that T21 in mice results in anemia, while human T21 is associated with polycythemia, and T21 combined with the *Gata1s* mutation in mice does not recapitulate human TAM (29–31).

In vitro pharmacologic inhibition of DYRK1A has been shown to augment maturation and polyploidization in human neonatal cord blood– and iPSC-derived megakaryocytes through modulation of megakaryoblastic leukemia 1 (MKL1) levels that drive adult megakaryopoiesis (21). Elagib et al. showed that DYRK1A inhibition with harmine or EHT 1610 did not enhance megakaryocyte proliferation, and in fact, decreased the overall number of cells by approximately 2-fold (21). An explanation for these seemingly differing effects of the loss of DYKR1A between Elagib et al.’s and our studies is that our cells have an extra copy of chromosome 21 and the proliferative effect and maturation arrest of DYRK1A knockout was only observed in the presence of GATA1s. Alternatively, the effects of harmine and EHT 1610 may not be specific to DYRK1A only and other related kinases may be affected, including other DYRK family members or CDC-like kinase 1 (CLK1) that cooperate with CDK4 to promote proliferation (47, 48). We observed a significant decrease in proliferative capacity of iPSC-derived megakaryocytes with DYRK1A knockout treated with either harmine or EHT 1610, suggesting off-target or toxic effects of these small molecules. Observed differences highlight the potential gene dosage– and cell context–dependent effects of DYRK1A.

DYRK1A has been shown to regulate the cell cycle and inhibit proliferation through the cyclin D/CDK-Rb/E2F pathway in nonhematopoietic cell types, including neural, cardiac, and fibroblast (19, 27, 28, 49). For example, DYRK1A interacts with all 3 D-type cyclins to inhibit Rb/E2F signaling and proliferation in murine cardiomyocytes (19). Consistent with those findings, we found that DYRK1A loss increases D-type cyclin expression, Rb phosphorylation, and other cell cycle–related gene expression in both wtGATA1 and GATA1s iPSC–derived megakaryocytes. However, increased megakaryocyte and myeloid proliferation was observed only in the context of GATA1s due to the ability of wtGATA1 to restrain cell proliferation. This unexpected finding suggests that DYRK1A may not be a driver for leukemogenesis in children with DS and may instead offer protection in ML-DS with its antiproliferative effects.

Interestingly, both euploid and T21 megakaryocytes with GATA1s have increased DYRK1A protein expression compared with megakaryocytes expressing wtGATA1, suggesting that DYRK1A may be activated in these hyperproliferative cells to inhibit further cell cycle entry and cell proliferation. E2F1 has been shown to upregulate DYRK1A expression in Saos2 and Phoenix cells (50), providing a possible explanation for the increased expression of DYRK1A in T21/GATA1s megakaryocytes and supporting a direct relationship between activator E2Fs and DYRK1A in cell cycle regulation.

DYRK1A likely regulates megakaryocyte biology through additional mechanisms, including the DREAM complex, a master cell cycle coordinator that is closely related to E2F pathways (28, 40, 51). Our RNA-seq analysis showed increased gene enrichment for DREAM complex targets in the T21/GATA1s/DYRK1A^–/–/–^ megakaryocytes, possibly from DYRK1A loss that leads to DREAM complex disassembly and increased D-type cyclin expression to allow activator E2Fs to drive cell growth. In support of this, we observed an increase in the phosphorylation of the DREAM complex component p130 in T21/GATA1s/DYRK1A^–/–/–^ megakaryocytes, which facilitates disassembly of the complex to allow cell cycle entry (data not shown). Taken together, these results show that DYRK1A may regulate the cell cycle through both the Rb/E2F and DREAM complexes synergistically.

Genes on HSA21, and in particular those located in the DS critical region (DSCR), have been previously investigated for their potential role in DS leukemogenesis. The DSCR, located in the distal half of the long arm, contains not only *DYRK1A* but also *RUNX1*, *ETS2*, and *ERG* (15). Although these key transcription factors normally cooperate with wtGATA1 to regulate megakaryocytic gene expression, they are typically not overexpressed in ML-DS (12, 52). Different DS models have disagreed on the effect of *RUNX1* expression levels in T21 hematopoiesis (53); interestingly, however, a recent combined study of murine models and human-derived xenografts suggested that the leukemic blasts are characterized by disequilibrium between RUNX1 isoforms (54). Meanwhile, overexpression of *ETS2* and *ERG* has been found in cell line and murine models to cooperate with mutated or knocked-down *GATA1* to induce expansion of the megakaryocytic compartment (52, 55, 56), suggesting that the gene dosage of the DSCR, including *DYRK1A*, contributes to TAM and ML-DS phenotypes but may not be sufficient.

Recent, focused studies on likely “third hits” in ML-DS have addressed the role of mutations in the cohesin complex. Contributions of mutations in *SMC3*, which encodes a member of the cohesin complex, and in *MPL*, which encodes the transmembrane TPO receptor, were studied in a human iPSC system (57). This model found that while gain-of-function mutations in *MPL* on a T21/GATA1s background impaired platelet maturation only, *SMC3* haploinsufficiency increased colony-forming potential, enhanced proliferation, and impaired differentiation of megakaryocytes, similar to our findings in T21/GATA1s/DYRK1A^–/–/–^ cells. Loss-of-function mutations in *STAG2*, another cohesin complex gene, was found in a human fetal liver xenograft model in mice to act synergistically with *GATA1s* mutations to drive engraftment and leukemic blast production (58). Taken together, these studies suggest that sequential genetic events cooperate in the evolution toward ML-DS.

While transformation of DS hematopoietic cells has been studied extensively, the contribution of specific HSA21 genes and the multistep process has been difficult to study, in part due to a paucity of appropriate human cell models. Moreover, while studies often focus on 1 particular HSA21-encoded gene, it is likely that gene dosage imbalance of several genes contributes to TAM and ML-DS. Human iPSC and genome editing technology has facilitated the development of cellular models to identify the roles of HSA21-encoded genes and other acquired mutations in ML-DS pathogenesis (57, 59). A limitation of these and the present study is that our differentiation protocols primarily recapitulate the first early wave of hematopoiesis that occurs in the yolk sac, termed primitive, while TAM and ML-DS arise in the fetal and postnatal definitive stages of blood ontogeny, respectively. Our findings may be context specific, but the use of isogenic human T21 iPSCs with and without GATA1s to examine the role of DYRK1A in megakaryocyte precursors facilitated our findings that unraveled the cell type–specific interplay between GATA1s and DYRK1A. These studies suggest that small molecule inhibitors of DYRK1A may not have therapeutic application for ML-DS and that other strategies need to be explored for targeted therapy.

## Methods

### Gene targeting.

The CRISPR/Cas9 nuclease system was used to target all isoforms of human DYRK1A in isogenic T21 iPSC lines with wtGATA1 or GATA1s that were previously generated by our laboratory (32, 33, 60). gRNAs were designed to target exon 3 of *DYRK1A* to generate iPSC clones with gene knockout (Supplemental Figure 2, A and B). Successful editing was confirmed by TOPO cloning, Western blotting, and/or quantitative PCR (qPCR) for all lines. Sequences of gRNA, PCR, and qPCR screening primers (Integrated DNA Technologies) are shown in Supplemental Table 1. Targeted iPSC lines with DYRK1A knockout are shown in Supplemental Table 2.

### Hematopoietic differentiation of iPSCs.

iPSC culture and hematopoietic differentiation by EB formation were performed as previously described (32, 34). CD43^+^ hematopoietic progenitor cells released by the EBs by day 7 of differentiation were collected for analysis or subsequent single-lineage liquid culture in serum-free differentiation (SFD) media supplemented with ascorbic acid (50 μg/mL), monothioglycerol (0.4 mM), and SCF (50 ng/mL) and with EPO (2 U/mL), TPO (50 ng/mL), or GM-CSF (10 ng/mL) and IL-3 (10 ng/mL) for erythroid, megakaryocytic, or myeloid differentiations, respectively. All cytokines except EPO (Amgen) and basic fibroblast growth factor (bFGF, Invitrogen) were purchased from R&D Systems. On days 4–6 of single-lineage culture, cells were collected for RNA-seq, flow cytometric analysis, fold-change calculation, and functional studies, including EdU, Ki67, PAC-1 binding, and Annexin V.

### Colony formation assay.

CD43^+^ hematopoietic progenitor cells were seeded in MethoCult H4230 (STEMCELL Technologies) with EPO (5 U/mL), IL-3 (10 ng/mL), SCF (50 ng/mL), GM-CSF (10 ng/mL), N2 supplement (Gibco, 100×), and B27 supplement (Gibco, 50×) at 500 or 1,000 cells/mL. Erythroid (CFU-E) and myeloid (CFU-GM) colonies were scored at 10–12 days. For CFU-Mk assays, cells were seeded in MegaCult-C collagen and media with cytokines (STEMCELL Technologies, 04961) according to the manufacturer’s instructions, with additional SCF (50 ng/mL) and N2 and B27 supplements to support colony formation. Colonies were dehydrated, fixed, and stained with anti-GPIIb/IIIa antibody (STEMCELL Technologies, 04803) according to the manufacturer’s instructions after 12 days of culture.

### Flow cytometric analysis.

Flow cytometric analysis was performed on a FACSCanto II or LSRFortessa (both BD Biosciences), as previously described (32). Antibodies used for surface marker staining are shown in Supplemental Table 3. Flow cytometry data and MFI were analyzed using FlowJo 10 (Tree Star, Inc.).

### Cell proliferation assays.

EdU incorporation was measured using the Click-iT Plus EdU Flow Cytometry Assay Kit (Invitrogen, C10632) according to the manufacturer’s instructions. Ki67 staining was performed per BioLegend’s Ki67 Flow Cytometry Staining Protocol after surface marker staining. FxCycle or Dyecycle Violet Stain (Invitrogen) was used for DNA content staining. Flow cytometry was performed to measure the percentages of EdU- and Ki67-positive megakaryocytes gated on the CD41^+^CD42b^+^ or CD42b^+^ population, respectively.

### Megakaryocyte activation assay.

PAC-1 binding assays for megakaryocyte activation were performed as previously described (61). Briefly, 0.5 × 10^6^ iPSC-derived megakaryocytes were resuspended in 100 μL Tyrode’s salt solution (Sigma-Aldrich) with 0.1% BSA and CD41a, CD42b, and PAC-1 antibodies, with or without thrombin (Sigma-Aldrich) stimulation, and incubated for 10 minutes at room temperature followed by 10 minutes on ice. Samples were then diluted with 450 μL Tyrode’s salt solution with 0.1% BSA and immediately analyzed by flow cytometry.

### Annexin V assay.

Day 5 or 6 iPSC-derived megakaryocytes were stained for CD41a and CD42b, and then resuspended in 100 μL Annexin V Binding Buffer (BioLegend) with 5 μL anti–Annexin V and 5 μL 7-AAD (BioLegend). After incubation for 20 minutes at room temperature in the dark, cells were diluted with 400 μL Annexin V Binding Buffer and immediately analyzed by flow cytometry.

### Cloning and lentiviral transduction.

The human *DYRK1A* coding sequence was cloned into the HMD lentiviral vector containing GFP to generate HMD-DYRK1A. Viral particles were generated in 293T cells by cotransfection of HMD empty vector or HMD-DYRK1A with VSV-G envelope and PAX2 packaging vectors using Lipofectamine 2000 (Thermo Fisher Scientific). Viral supernatants were collected 48 hours after transfection and concentrated 50-fold (Lenti-X Concentrator, TaKaRa). For lentiviral transduction, 2 × 10^6^ CD43^+^ hematopoietic progenitor cells in SFD media with SCF (50 ng/mL), TPO (50 ng/mL), HEPES (10 mM), 50 μL of concentrated virus, and polybrene (2 ng/mL) were combined in 1 well of a 6-well plate followed by centrifugation at 1450*g* for 90 minutes at 25°C. Cells were then resuspended to 0.5 × 10^6^/mL in SFD media with SCF and TPO. Media were changed after overnight transduction.

### RNA-seq.

Day 7 hematopoietic progenitor cells and day 4 iPSC-derived megakaryocytes were sorted for CD41^+^CD235^+^ and CD41^+^CD42b^+^, respectively. Total RNA was extracted from these sorted cells using the RNeasy Micro Kit (Qiagen) according to the manufacturer’s instructions. RNA quality and integrity were measured by spectrophotometer (Nanodrop, Thermo Fisher Scientific) and capillary electrophoresis separation (LabChip GX Bioanalyzer, PerkinElmer). Ribodepletion (Ribo-Zero Plus, Illumina), library preparation (TruSeq Stranded Total RNA with Illumina Ribo-Zero Plus rRNA Depletion, Illumina), and sequencing (NovaSeq 6000 platform and S2 Reagent Kit v1.5, Illumina) were performed by the Center for Applied Genomics (Children’s Hospital of Philadelphia).

The RNA-seq data in .fastq file format were aligned to the *Homo* *sapiens* reference genome GRCh38 and transcriptome using the STAR (Spliced Transcripts Alignment to a Reference) program (https://github.com/alexdobin/STAR). STAR was run in the 2-pass mode to align reads to the reference genome and transcriptome, as well as novel splice junction sites detected by multiple experimental samples. Aligned reads in .bam files were loaded into R (https://www.r-project.org) and mapped to the sense strand of known exons and genes. A gene-level read count matrix based on uniquely mapped and properly paired reads was used for analysis of differential expression after reads of technical replicates were pooled together. The pooled read counts were converted to fragment per kilobase per million reads (FPKM) to represent gene expression levels.

The R/Bioconductor DESeq2 package was used to test differential gene expression between DYRK1A-WT and -knockout samples. The top differentially expressed genes (DEGs) were selected using a log_2_(fold-change) of greater than 2.0, a standard FDR of 0.25, and a *P* value of less than 0.01. GSEA was run on the identified DEGs using gene set collections obtained from BioSystems (https://pubmed.ncbi.nlm.nih.gov/19854944), KEGG (https://www.genome.jp/kegg), and MSigDb v7.4 (https://www.gsea-msigdb.org/gsea/msigdb). All genes with detectable expression in the RNA-seq samples were used as background to test enrichment of predefined gene sets.

### qRT-PCR.

Total RNA from day 4 iPSC–derived megakaryocytes was extracted using the RNeasy Micro kit, and cDNA was prepared using the iScript cDNA Synthesis Kit (Bio-Rad) according to the manufacturer’s instructions. qRT-PCR was performed on the ViiA7 Real-Time PCR System (Applied Biosystems) using Power SYBR-Green PCR Master Mix (Life Technologies) according to the manufacturer’s instructions. Gene expression was normalized to *GAPDH*, and relative quantification was determined by the comparative Ct method. Primers used for qPCR are shown in Supplemental Table 4.

### Western blotting.

Cells were lysed in Pierce RIPA buffer (Thermo Fisher Scientific) supplemented with 1% HALT protease and phosphatase inhibitor cocktail (Thermo Fisher Scientific). Extracted protein samples were normalized using the Pierce BCA Protein Assay Kit (Thermo Fisher Scientific), fractionated on Bolt 4%–12% Bis-Tris gradient gels (Invitrogen), and transferred to 0.2-μm PVDF membranes (GE Healthcare). Primary antibodies and HRP-conjugated secondary antibodies are shown in Supplemental Table 5. SuperSignal West Femto or SuperSignal West Atto chemiluminescent substrate (Thermo Fisher Scientific) was used for imaging. Fiji (ImageJ) was used to analyze band intensity.

### Statistics.

All statistical analysis was performed using GraphPad Prism 9. Data were analyzed by 2-tailed, unpaired *t* test for comparison of 2 conditions or ordinary 1-way ANOVA for multiple comparisons. All data are summarized as the mean ± standard error of the mean (SEM). A *P* value of less than 0.05 was considered significant: *P ≤ 0.05, **P ≤ 0.01, ***P ≤ 0.001, ****P ≤ 0.0001.

### Study approval.

All patients or legal guardians provided written informed consent to participate in this study. The institutional review boards at the Children’s Hospital of Philadelphia and the University of Pennsylvania approved the study protocols.

### Data availability.

RNA-seq data reported in this paper have been deposited in the NCBI’s Gene Expression Omnibus with GEO series accession number GSE243702. Data values for all graphs are included in the Supporting Data Values XLS file.

## Author contributions

YTS designed research studies, performed experiments, acquired data, interpreted the data, and wrote the manuscript. KT interpreted data and wrote the manuscript. HHA, YX, and CH performed experiments and edited the manuscript. PAG provided reagents. ZZ analyzed the RNA-seq data. PG and DLF designed research studies, interpreted the data, and edited the manuscript. STC designed research studies, interpreted the data, and wrote the manuscript.

## Supplementary Material

Supplemental data

Supporting data values

## Version 1 10/31/2023

In-Press Preview

## Version 2 12/08/2023

Electronic publication
