# Supplementary material for: Synergistic roles of DYRK1A and GATA1 in trisomy 21 megakaryopoiesis
Source: JCI Insight. 2023 Oct 31;8(23):e172851. doi: 10.1172/jci.insight.172851 (PMC10895998; doi:10.1172/jci.insight.172851)
Supplement: Supplemental data [file jciinsight-8-172851-s096.pdf]

Supplemental Table 1. CRISPR/Cas9 targeting guide RNAs, oligonucleotides for homologous recombination, and PCR primers

TMD patient 8	
gRNA (5'-3')	CACTTATGTTTGGCTGGCGACGG
PCR primers	F: GAGACTTCAGCATGCAAACC<br>R: AAACTGCCATTCCAATAGTC
TMD patient 145	
gRNA (5'-3')	CGCCAGCATTCCCCAAACCAGG &<br>CACCGTCTACGTTGAGTTTTGG
PCR primers to detect *DYRK1A* large deletion	F: TCATGGTTCATCCTACCCGT<br>R: GTTTTCCTAGCCCGCTGTAGAC
qPCR primers to detect *DYRK1A* copy number	F: CCAAACCTTCCGTGACCCAG<br>R: TAGAATCGTCTCCCTGGCCC
qPCR primers to detect *DYRK1A* large deletion	F: CGTCGCCAGCCAAACATAAG<br>R: TCAGGCATCACCTGGTTAGT

Supplemental Table 2. *GATA1* and *DYRK1A* characterization of induced pluripotent stem cell clones

**TMD patient**	***GATA1***	***DYRK1A***	
TMD patient 8 (T21)	wtGATA1	-/-/-	Clone 1
		-/-/-	Clone 2
	GATA1 g.4652G>T (GATA1s)	-/-/-	Clone 1
		-/-/-	Clone 2
		-/-/-	Clone 3
TMD patient 8 (Euploid)	GATA1 g.4652G>T (GATA1s)	-/-	Clone 1
TMD patient 145	GATA1 c.3_4insG (GATA1s)	-/-/-	Clone 1
		-/-/-	Clone 2

Supplemental Table 3. Flow cytometry antibodies

Antibody	Manufacturer	Catalog #	Dilution
SSEA-3<br>Alexa Fluor 488	BioLegend<br>Clone MC-631	330306	1:200
SSEA-4<br>Alexa Fluor 647	BioLegend<br>Clone MC-813-70	330408	1:200
Tra-1-60<br>Alexa Fluor 488	BioLegend<br>Clone TRA-160-R	330614	1:40
Tra-1-81<br>Alexa Fluor 647	BioLegend<br>Clone TRA-1-81	330706	1:40
KDR (CD309)<br>Alexa Fluor 647	BioLegend<br>Clone 7D4-6	359910	1:100
CD31<br>FITC	BD Biosciences<br>Clone WM59	555445	1:20
CD34<br>APC	BioLegend<br>Clone 561	561209	1:100
CD43<br>FITC	BD Biosciences<br>Clone 1G10	555475	1:20
CD41a<br>PE	BD Biosciences<br>Clone HIP8	555467	1:20
CD41a<br>PE/Cy7	BioLegend<br>Clone HIP8	303718	1:400
CD42a<br>FITC	BD Biosciences<br>Clone ALMA.16	558818	1:20
CD42b<br>APC	BD Biosciences<br>Clone HIP1	551061	1:40
CD42b<br>FITC	BD Biosciences<br>Clone HIP1	555472	1:40
CD235a<br>APC	BD Biosciences<br>Clone GA-R2	551336	1:5000
CD71<br>FITC	BD Biosciences<br>Clone M-A712	555536	1:20
CD45<br>PE	BD Biosciences<br>Clone HI30	555483	1:20

CD18<br>APC	BD Biosciences<br>Clone 6.7	551060	1:20
PAC-1<br>FITC	BD Biosciences<br>Clone PAC-1	340507	1:20
Ki67<br>APC	BioLegend<br>Clone Ki-67	350514	1:100
Annexin V<br>FITC	BD Biosciences	556420	1:20
7-AAD	BioLegend	420404	1:200
FxCycle Violet	Invitrogen	F10347	1:1000
DyeCycle Violet	Invitrogen	V35003	1:1000

Supplemental Table 4. Real-time qPCR primers (5'-3')

hGAPDH	F: GTCAGTGGTGGACCTGACCT<br>R: TGAGCTTGACAAAGTGGTCG
hCyclin D1	F: TCTACACCGACAACTCCATCCG<br>R: TCTGGCATTTTGGAGAGGAAGTG
hCyclin D2	F: GAGAAGCTGTCTCTGATCCGCA<br>R: CTTCCAGTTGCGATCATCGACG
hGATA2	F: CAGCAAGGCTCGTTCCTGTTCA<br>R: ATGAGTGGTCGGTTCTGCCCAT
hCyclin A2	F: AACCATTGGTCCCTCTTGATTAT<br>R: TCCTCATGGTAGTCTGGTACTT
hCDC6	F: GGAGATGTTCGCAAAGCACTGG<br>R: GGAATCAGAGGCTCAGAAGGTG
hCDC25A	F: TCTGGACAGCTCCTCTCGTCAT<br>R: ACTTCCAGGTGGAGACTCCTCT
hE2F8	F: GAGGCTCAAAGAGGGCAAGCAT<br>R: ATGAGCACTGCGTGAGAGGGAT
hMCM2	F: TGCCAGCATTGCTCCTTCCATC<br>R: AAACTGCGACTTCGCTGTGCCA
hCHK1	F: GTGTCAGAGTCTCCCAGTGGAT<br>R: GTTCTGGCTGAGAACTGGAGTAC
hPF4	F: CCCACTGCCCAACTGATAG<br>R: GCAAATGCACACACGTAGG
hVWF	F: TCCCCTGTCTCATCGGA<br>R: GCACTCCAGGTCATAGTTCTG
hGP9 (CD42a)	F: TACCTGCCGCGCCCTGGAAAC<br>R: CACGGACTGAAGGCTGTTGTTG
hSELP (CD62P)	F: TCCGCTGCATTGACTCTGGACA<br>R: CTGAAACGCTCTCAAGGATGGAG

Supplemental Table 5. Western blot antibodies

	Antibody	Manufacturer	Catalog #	Dilution
Primary antibodies	DYRK1A	Cell Signaling Technology	8765	1:750
		Abcam	ab65220	1:600
	Cyclin D1	Cell Signaling Technology	55506	1:1000
	Cyclin D2	Cell Signaling Technology	3741	1:1000
	Cyclin D3	Cell Signaling Technology	2936	1:2000
	Phospho-Cyclin D2 (Thr280)	Aviva Systems Biology	OAAJ06410	1:500
	Phospho-Cyclin D3 (Thr283)	Cell Signaling Technology	53966	1:1000
	Cyclin A2	Cell Signaling Technology	67955	1:1000
	Cyclin B1	Cell Signaling Technology	4138	1:1000
	CDK1	Cell Signaling Technology	9116	1:1000
	β-actin	Abcam	ab6276	1:20000
	Hypophospho-Rb	BD Biosciences	554164	1:500
	Phospho-Rb (Ser780)	Cell Signaling Technology	8180	1:1000
	Phospho-Rb (Ser807/811)	Cell Signaling Technology	9308	1:1000
	Rb	BD Biosciences	554136	1:500
	GATA1	Cell Signaling Technology	4589	1:1000
	GATA2	Abcam	ab109241	1:1000
	CDK4	Cell Signaling Technology	12790	1:1000
	GAPDH	Abcam	ab181602	1:10000
Secondary antibodies	Mouse IgG (HRP-conjugated)	Invitrogen	31430	1:20000
	Rabbit IgG (HRP-conjugated)	Cell Signaling Technology	7074	1:10000

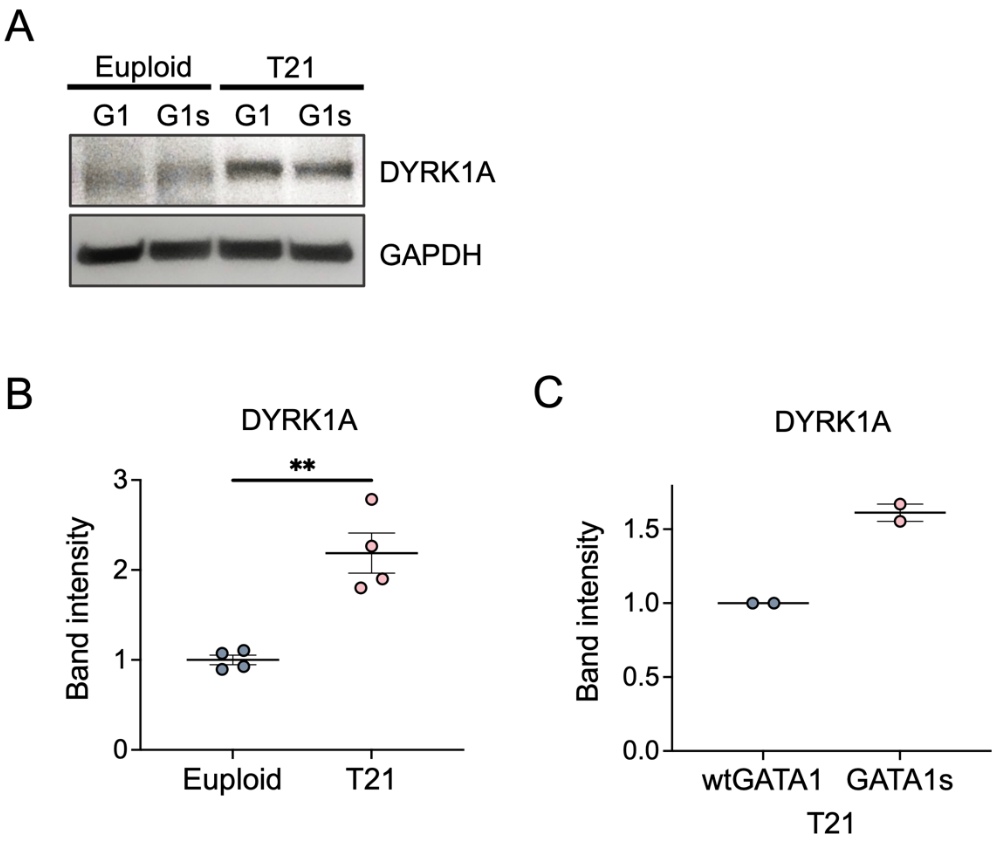

Supplemental figure 1. DYRK1A is overexpressed in human trisomy 21 cells.
(A) Representative Western blot for DYRK1A expression in isogenic undifferentiated iPSCs from euploid or T21 iPSCs, with wild-type GATA1 (G1) or GATA1short (G1s). DYRK1A expression quantified by Western blot band intensity for **(B)** Euploid and T21 iPSC and **(C)** day 6 T21/wtGATA1 and T21/GATA1s megakaryocytes. Bands normalized to housekeeping gene. n = 4 and 2 independent experiments per genotype for **(B)** and **(C)**, respectively. Data represent the mean ± SEM. Statistical significance was determined by 2-tailed, unpaired t-test. **$p \leq 0.01$.

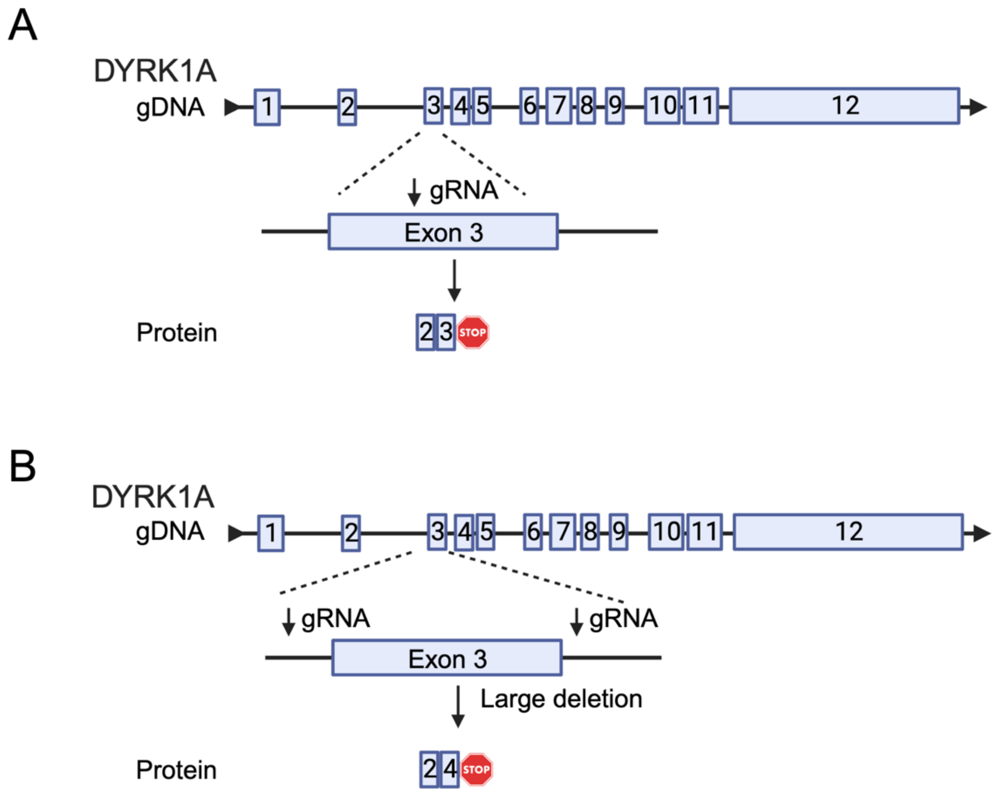

Supplemental figure 2. CRISPR/Cas9 targeting of *DYRK1A* in T21 iPSCs.

CRISPR/Cas9 targeting strategy for *DYRK1A* knockout in T21/wtGATA1 and T21/GATA1s iPSCs. gRNAs were designed to **(A)** target exon 3 of human *DYRK1A* to introduce an insertion-deletion in patient 1, or **(B)** target introns 2 and 3 of human *DYRK1A* to generate a large deletion that includes exon 3 in patient 2.

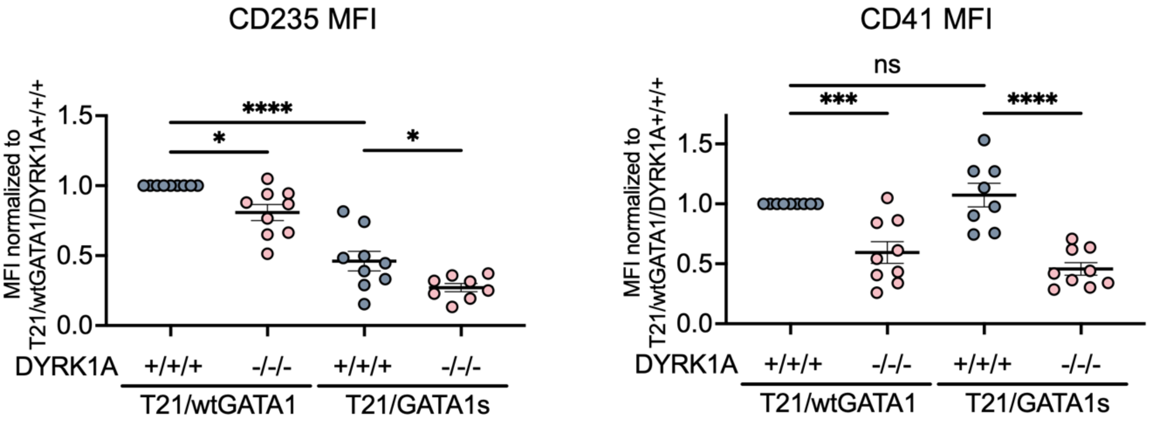

Supplemental figure 3. DYRK1A loss shows aberrant hematopoietic progenitors regardless of GATA1 status. Mean fluorescence intensity (MFI) for CD41 and CD235 for day 7 hematopoietic progenitors gated on $CD43^{+}41^{+}235^{+}$ and normalized to MFI for T21/wtGATA1/$DYRK1A^{+/+/+}$. n = 8-9 independent experiments per genotype. Data represent the mean ± SEM. Statistical significance was determined by ordinary one-way ANOVA. ns, non-significant, *$p \leq 0.05$, ***$p \leq 0.001$, ****$p \leq 0.0001$.

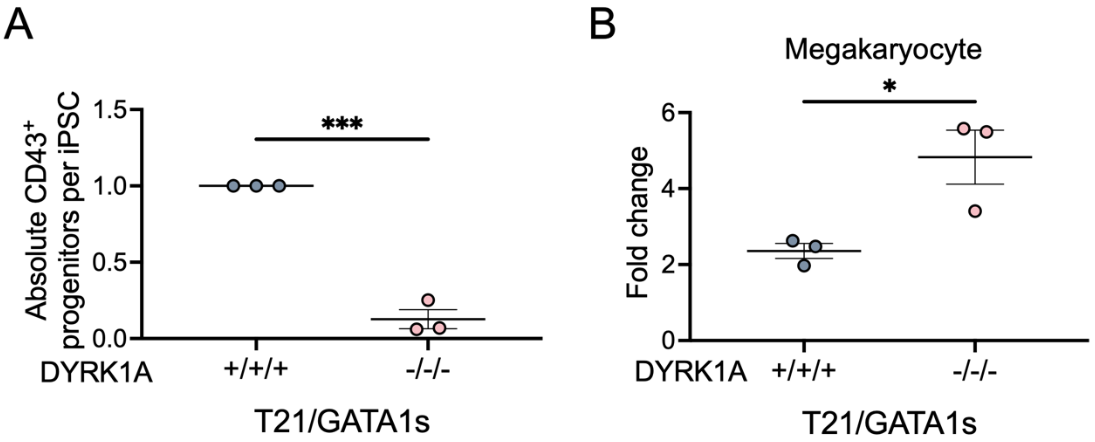

Supplemental figure 4. Hematopoietic phenotype of independently generated iPSC clones from a distinct patient with T21/GATA1s. (A) Absolute CD43+ progenitor number generated per iPSC undergoing differentiation. **(B)** Fold change of $CD41^{+}42^{+}$ megakaryocytes differentiated in liquid culture for 6 days from T21/GATA1s iPSC-derived CD43+ progenitors with DYRK1A wild-type (+/+/+) or knockout (-/-/-). n =3 independent experiments per clone. Data represent the mean ± SEM. Statistical significance was determined by 2-tailed, unpaired t-test. *$p \le 0.05$, ***$p \le 0.001$.

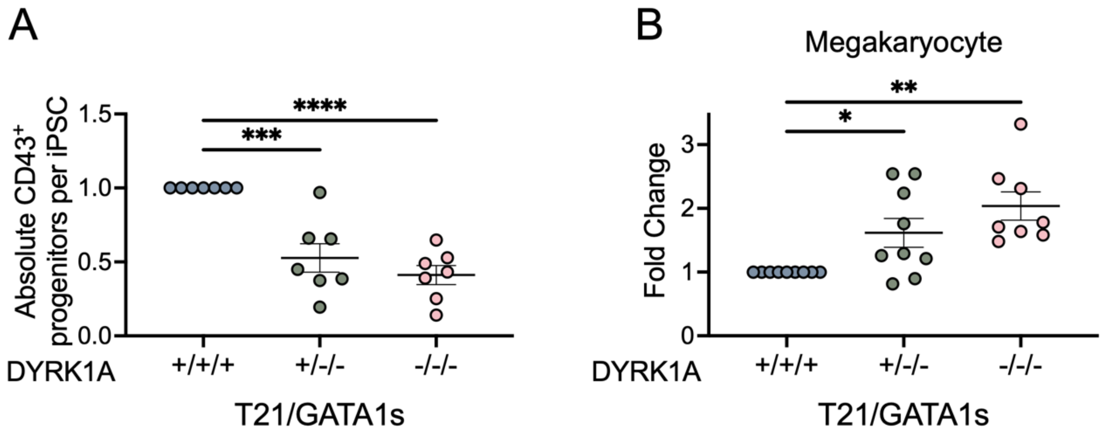

Supplemental figure 5. DYRK1A knockdown showed intermediate phenotype compared to DYRK1A knockout. (A) Absolute CD43+ progenitor yield on day 7 of EB differentiation, normalized to starting DYRK1A$^{+/+/+}$ iPSC number. **(B)** Fold change from T21/GATA1s iPSC-derived CD43+ hematopoietic progenitors with DYRK1A wild-type, knockdown or knockout, differentiated in liquid culture for 6 days to support megakaryocytes (CD41^{+}42^{+}), normalized to starting DYRK1A$^{+/+/+}$ CD43+ progenitors. Each column represents an individual iPSC clone. *n* = 7-9 independent experiments per clone. Data represent the mean ± SEM. Statistical significance was determined by ordinary one-way ANOVA. *$p \leqslant 0.05$, **$p \leq 0.01$, ***$p \leq 0.001$, ****$p \leq 0.0001$.

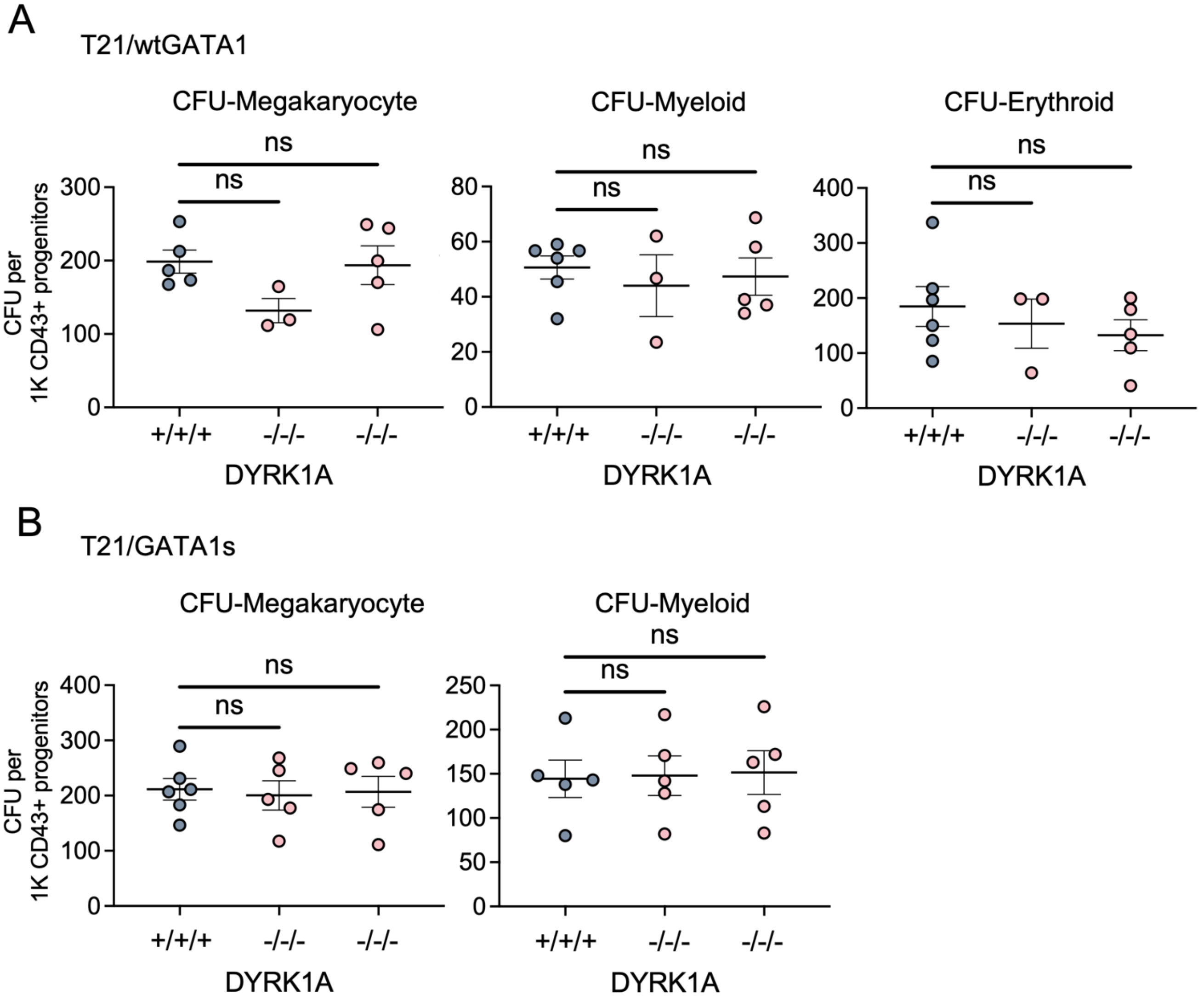

Supplemental figure 6. DYRK1A knockout does not affect hematopoietic colony formation. Colony-forming unit (CFU) assays were performed on CD43$^+$ progenitors derived from **(A)** T21/wtGATA1 and **(B)** T21/GATA1s iPSCs with DYRK1A wild-type (+/+/+) or knockout (-/-/-). Each column represents an individual iPSC clone. *n* = 3-6 independent experiments per clone. Data represent the mean ± SEM. Statistical significance was determined by ordinary one-way ANOVA. ns, non-significant.

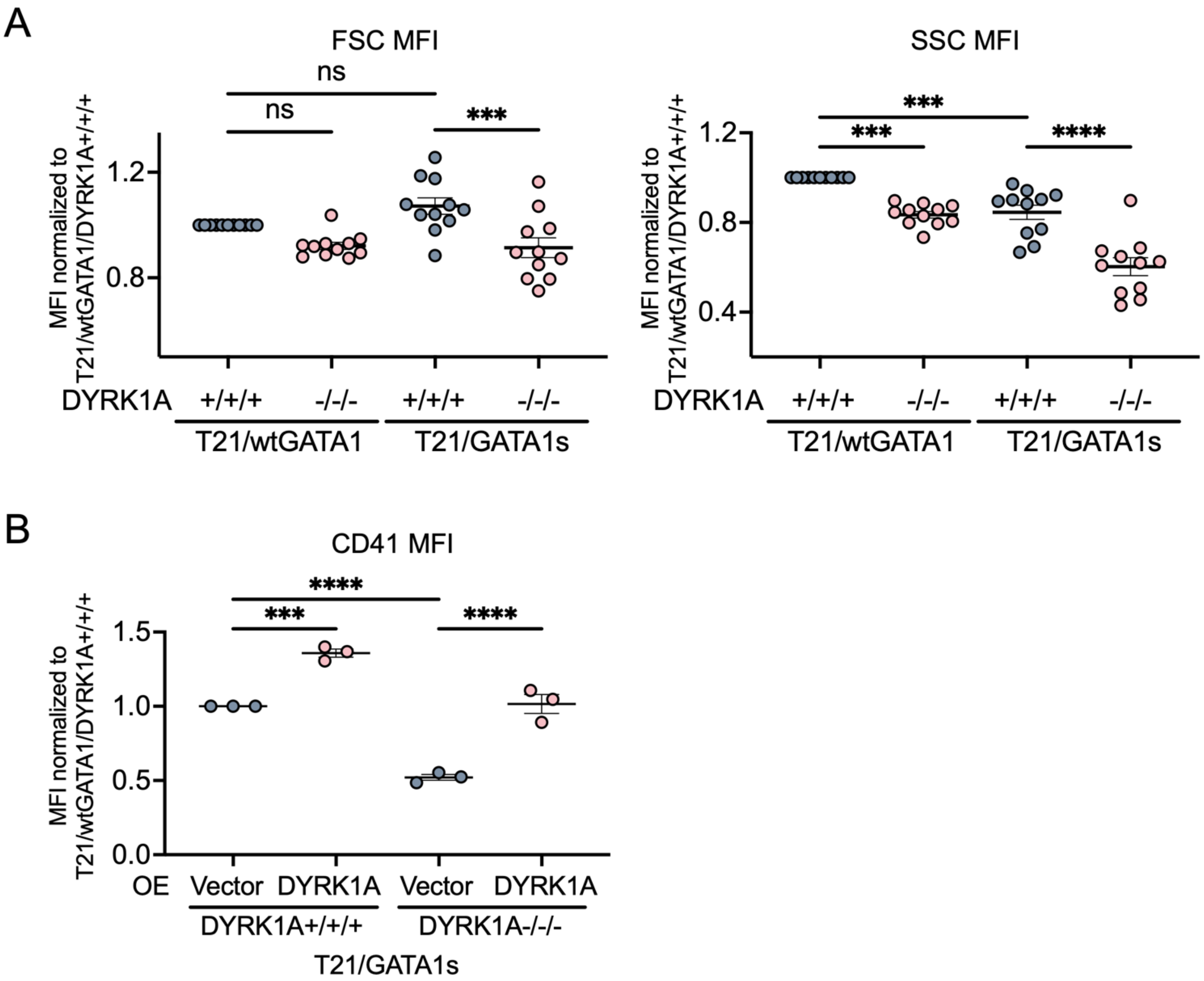

Supplemental figure 7. DYRK1A knockout impairs megakaryocyte maturation in T21/GATA1s cells. (A) Forward scatter (FSC) and side scatter (SSC) for day 5 to 6 $CD41^{+}42b^{+}$ megakaryocytes. *n* = 11 independent experiments per clone. **(B)** CD41 mean fluorescence intensity (MFI) of T21/GATA1s $DYRK1A^{+/+/+}$ and $DYRK1A^{-/-/-}$ megakaryocytes assayed on day 5 to 6 of lineage-specific liquid culture after lentiviral overexpression (OE) of DYRK1A compared to vector alone. *n* = 3 independent experiments. Data represent the mean ± SEM. Statistical significance was determined by ordinary one-way ANOVA. ns, non-significant, ***$p \leq 0.001$, ****$p \leq 0.0001$.

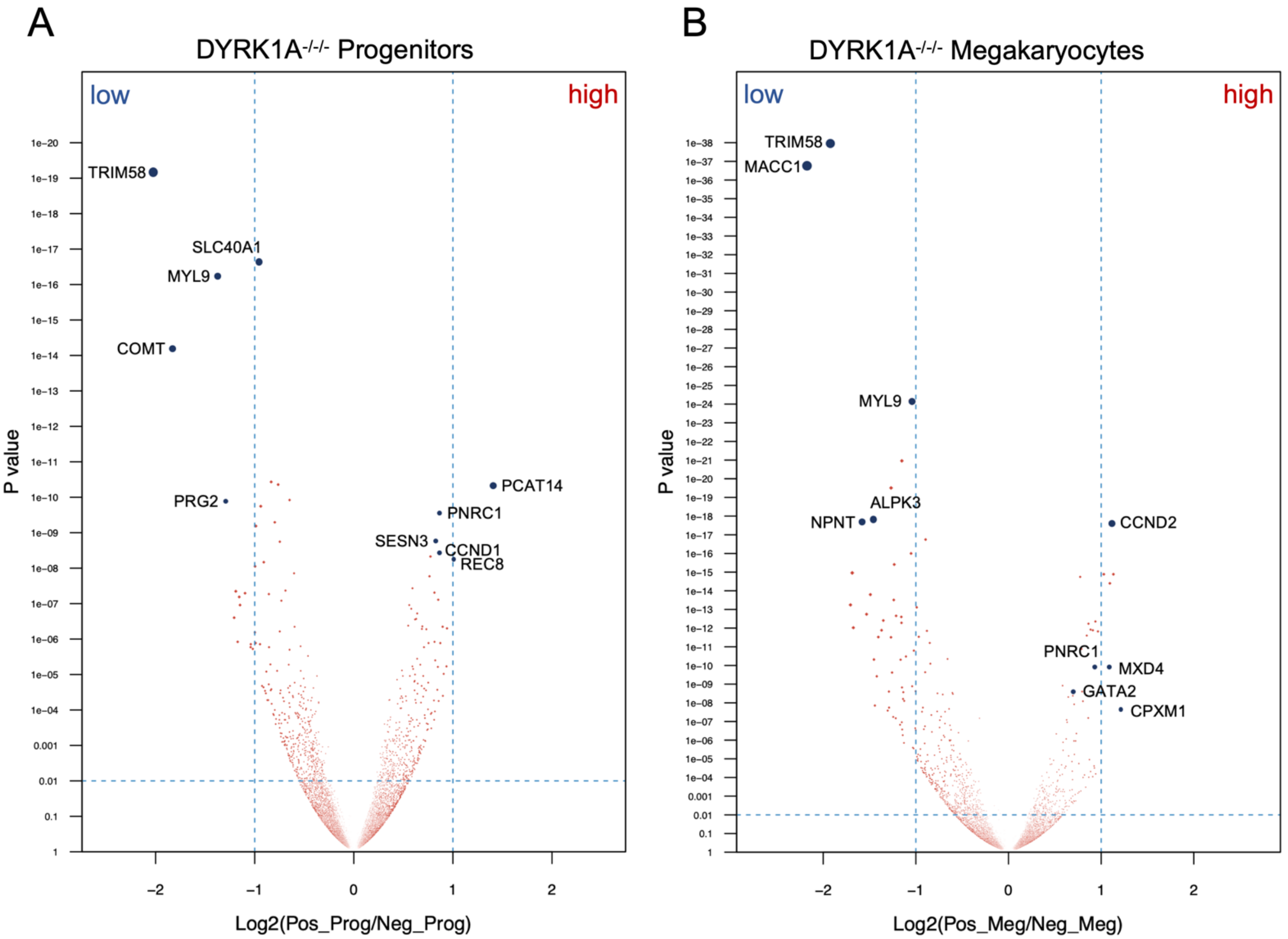

Supplemental figure 8. Volcano plots showing differential gene expression for T21/GATA1s/DYRK1A$^{-/-/-}$ compared with T21/GATA1s/DYRK1A$^{+/+/+}$ in sorted progenitors **(A)** and day 4 megakaryocytes **(B)**.
Each dot represents a gene, top 5 up and down-regulated genes in DYRK1A$^{-/-/-}$ compared to DYRK1A$^{+/+/+}$ are labeled in blue.

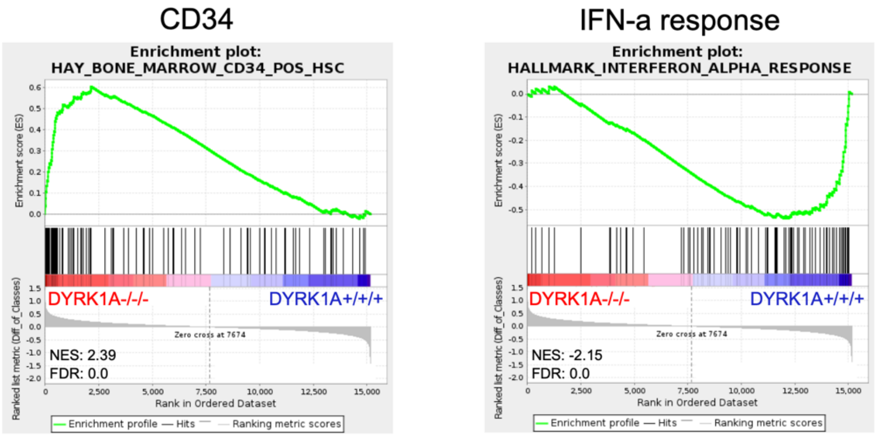

Supplemental figure 9. RNA-seq analysis shows enhanced CD34 cell properties and decreased interferon signaling in T21/GATA1s/DYRK1a$^{-/-/-}$ progenitors. Selected gene set enrichment analysis (GSEA) plots of RNA-sequencing data from sorted T21/GATA1s/DYRK1A$^{+/+/+}$ and DYRK1A$^{-/-/-}$ CD43^{+}CD41^{+}235^{+} hematopoietic progenitors.

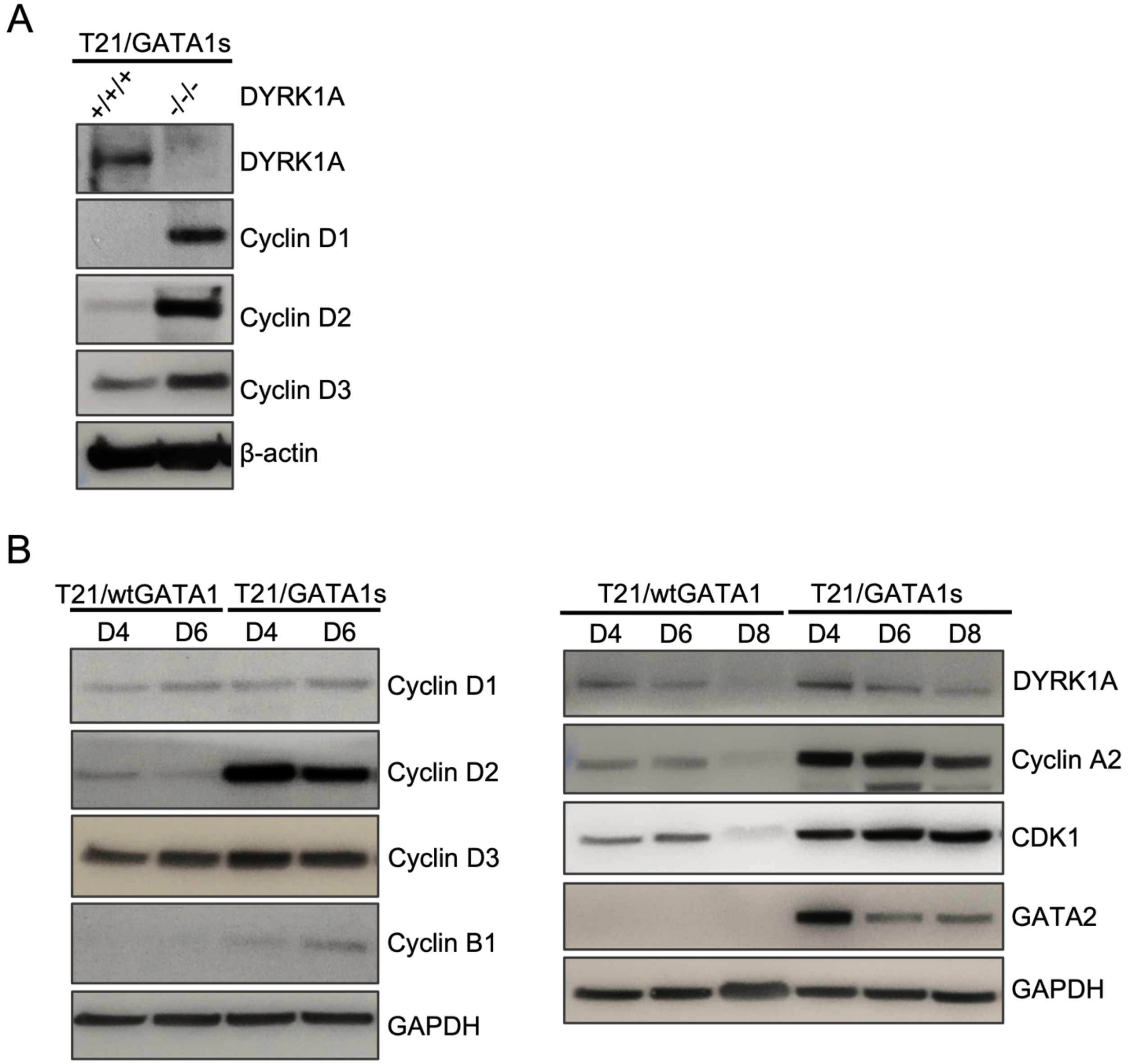

Supplemental figure 10. Cell cycle protein expression in iPSC-derived megakaryocytes from independently generated iPSC clones from a distinct patient with T21/GATA1s. Representative western blot analysis of **(A)** D-type cyclins in T21/GATA1s megakaryocytes with DYRK1A wild-type (+/+/+) or knockout (-/-/-) on day 6 of liquid culture and **(B)** cell cycle proteins in isogenic T21/wtGATA1 and T21/GATA1s iPSC-derived megakaryocytes on days 4, 6, and 8 of lineage-specific liquid culture.

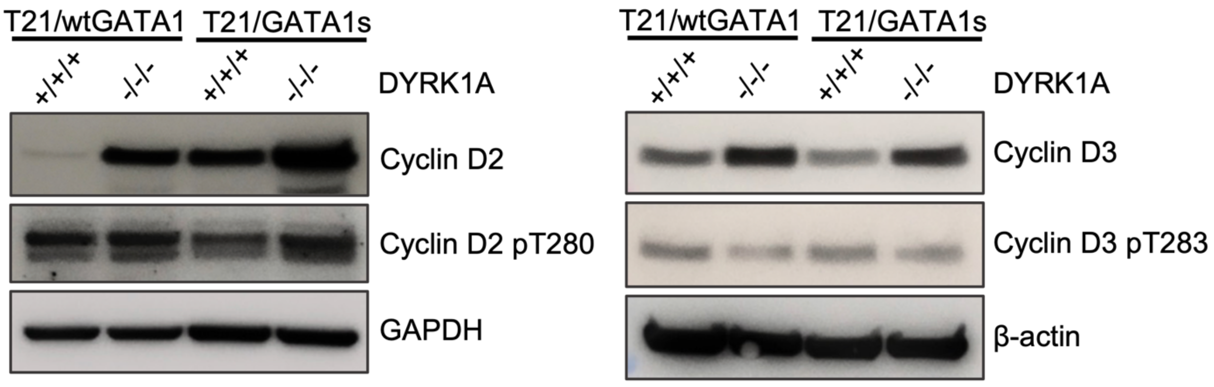

Supplemental figure 11. DYRK1A knockout decreases Cyclin D2 and Cyclin D3 phosphorylation level. Representative Western blot analysis of day 4 T21/wtGATA1 or T21/GATA1s megakaryocytes with DYRK1A wild-type (+/+/+) or knockout (-/-/-) for phosphorylated and total Cyclin D2 and Cyclin D3.
